# Direct measurements of neurosteroid binding to specific sites on GABA_A_ receptors

**DOI:** 10.1111/bph.16490

**Published:** 2024-07-08

**Authors:** Satyanarayana M. Chintala, Hiroki Tateiwa, Mingxing Qian, Yuanjian Xu, Fatima Amtashar, Zi-Wei Chen, Charles C. Kirkpatrick, John Bracamontes, Allison L. Germann, Gustav Akk, Douglas F. Covey, Alex S. Evers

**Affiliations:** 1 Department of Anesthesiology, Washington University School of Medicine, St. Louis, Missouri, USA; 2 Department of Anesthesiology and Intensive Care Medicine, Kochi Medical School, Kochi, Japan; 3 Department of Developmental Biology (Pharmacology), Washington University School of Medicine, St. Louis, Missouri, USA; 4 Taylor Family Institute for Innovative Psychiatric Research, St. Louis, Missouri, USA; 5 Department of Chemistry, Saint Louis University, St. Louis, Missouri, USA; 6 Department of Psychiatry, Washington University School of Medicine, St. Louis, Missouri, USA

**Keywords:** Fӧrster resonance energy transfer, GABA_A_ receptors, neuroactive steroids, neurosteroids, tryptophan quenching

## Abstract

**Background and Purpose:**

Neurosteroids are allosteric modulators of GABA_A_ currents, acting through several functional binding sites although their affinity and specificity for each site are unknown. The goal of this study was to measure steady-state binding affinities of various neurosteroids for specific sites on the GABA_A_ receptor.

**Experimental Approach:**

Two methods were developed to measure neurosteroid binding affinity: (1) quenching of specific tryptophan residues in neurosteroid binding sites by the neurosteroid 17-methylketone group, and (2) FRET between MQ290 (an intrinsically fluorescent neurosteroid) and tryptophan residues in the binding sites. The assays were developed using ELIC-α1GABA_A_R, a chimeric receptor containing transmembrane domains of the α_1_-GABA_A_ receptor. Tryptophan mutagenesis was used to identify specific interactions.

**Key Results:**

Allopregnanolone (3α-OH neurosteroid) was shown to bind at intersubunit and intrasubunit sites with equal affinity, whereas epi-allopregnanolone (3β-OH neurosteroid) binds at the intrasubunit site. MQ290 formed a strong FRET pair with W246, acting as a site-specific probe for the intersubunit site. The affinity and site-specificity of several neurosteroid agonists and inverse agonists was measured using the MQ290 binding assay. The FRET assay distinguishes between competitive and allosteric inhibition of MQ290 binding and demonstrated an allosteric interaction between the two neurosteroid binding sites.

**Conclusions and Implications:**

The affinity and specificity of neurosteroid binding to two sites in the ELIC-α1GABA_A_R were directly measured and an allosteric interaction between the sites was revealed. Adaptation of the MQ290 FRET assay to a plate-reader format will enable screening for high affinity agonists and antagonists for neurosteroid binding sites.

## 1 | INTRODUCTION

Neurosteroids (NS) are a family of steroids that are synthesized in the CNS and regulate neuronal excitability, largely by modulating the activity of GABA_A_ receptors (Alexander et al., 2021; Belelli & Lambert, 2005). NS with a 3α-hydroxyl group (3α-OH; e.g., allopregnanolone [AlloP] and pregnanolone [PREG]) are positive allosteric modulators (PAMs) of GABA_A_ receptors that enhance GABA-elicited chloride currents and thus reduce neuronal excitability. PAM-NS and their synthetic analogues have been clinically used as general anaesthetics (Kharasch & Hollmann, 2015; Manzella et al., 2022; Tateiwa & Evers, 2024), anti-depressants (Meltzer-Brody et al., 2018) and anti-epileptics (Knight et al., 2022). PAM-NS modulate GABA_A_ receptors by binding to specific sites on the protein. This was initially shown using site-directed mutagenesis with electrophysiological readout (Hosie et al., 2006). Subsequent direct identification of binding sites has been achieved using photoaffinity labelling (Z.-W. Chen et al., 2012, 2019), X-ray crystallography (Q. Chen et al., 2018; Laverty et al., 2017; Miller et al., 2017) and cryogenic electron microscopy (cryo-EM) (Sun et al., 2023). Direct measurement of reversible anaesthetic binding to these sites has not been feasible because NS are hydrophobic and bind with low affinity, making it difficult to separate free from bound ligand. This gap has limited the ability to determine binding affinities or kinetics and to develop high-throughput screening assays to identify high-affinity agonists and antagonists for NS binding sites. In this study, we describe fluorescence-based methods to directly measure NS binding.

The binding site underlying the PAM effect of NS was initially localized to an ‘intersubunit’ cleft between the β and α subunits in the cytoplasmic leaflet of the membrane-spanning α-helices of a heteromeric GABA_A_ receptor (Z.-W. Chen et al., 2012; Hosie et al., 2006). This binding site was confirmed by X-ray crystallographic studies detailing PAM-NS binding to pentameric chimeric proteins in which each subunit contains the transmembrane domain (TMD) of a GABA_A_ receptor α-subunit and the extracellular domain of a related pentameric ligand-gated ion channel (Q. Chen et al., 2018; Laverty et al., 2017; Miller et al., 2017) and more recently by cryo-EM studies of native murine heteropentameric GABA_A_ receptors (Sun et al., 2023). In this binding site, the 3α-OH of the NS forms a hydrogen bond with glutamine 242 (Q242), and the NS rings have hydrophobic interactions with tryptophan 246 (W246) in TM1 of the α-subunit. Mutations of either Q242 or W246 markedly reduce NS potentiation of GABA-elicited currents, consistent with the functional importance of this binding site (Akk et al., 2008; Hosie et al., 2006, 2007).

Photoaffinity labelling studies have identified two additional ‘intrasubunit’ sites for PAM-NS on α_1_β_3_ GABA_A_ receptors (Z.-W. Chen et al., 2019). AlloP binds between TM1 and TM4 in the extracellular leaflet of the α_1_-subunit. Mutations in this α_1_-intrasubunit binding site reduce PAM-NS potentiation of GABA_A_ receptors (Z.-W. Chen et al., 2019; L. Wang et al., 2022). AlloP also binds to a β_3_-intrasubunit site in the extracellular leaflet of the TMD between TM3 and TM4. Interestingly, this β_3_-intrasubunit site mediates inhibition of GABA-gated currents (Sugasawa et al., 2020) and thus antagonizes PAM-NS actions at the α_1_-intrasubunit and intersubunit sites. NS with a 3β-OH group, such as epi-allopregnanolone (Epi-AlloP), are negative allosteric modulators (NAM) of GABA_A_ receptors (M. Wang et al., 2002). These NS bind to both the α- and β-intrasubunit sites, where they act to promote receptor desensitization, but do not bind to the intersubunit site (Sugasawa et al., 2020). Notably, 3-sulfated NS such as pregnenolone sulfate represent another class of NAM-NS. The 3-sulfated NS act at binding sites on GABA_A_ receptors distinct from those occupied by 3-OH NS (Laverty et al., 2017; Legesse et al., 2023; M. Wang et al., 2002) and are not addressed in the current study.

The existence of multiple functional binding sites for NS on GABA_A_ receptors highlights the importance of determining the relative binding affinity of various NS analogues for each site and the potential therapeutic value of developing site-specific agonists and antagonists. All three of the identified NS binding sites on the GABA_A_ receptor are in close proximity to tryptophan residues. Tryptophan is intrinsically fluorescent with an excitation maximum near 280 nm and emission between 300 and 400 nm, thus providing a localized fluorescence energy ‘donor’ in each binding site that can be used to measure the proximity of bound NS molecules. In this study, we use two tryptophan proximity assays to measure NS binding: One assay is based on collisional quenching via photo-induced electron transfer (PET); PET requires direct contact between tryptophan and the quenching ligand and thus reports on distances < 5 Å. The second assay is based on Förster resonance energy transfer (FRET); the intensity of FRET signals is inversely proportional to the sixth power of the distance between the donor and NS acceptor, making these measurements extremely sensitive to molecular proximity. In the quenching assay we used the 17-methylketone of endogenous NS to quench tryptophan fluorescence. For the FRET assay, we synthesized a NS analogue with three conjugated double bonds in the steroid ring structure. This analogue, MQ290, replicates the PAM effect of NS on GABA_A_ receptors, has an absorbance maximum at 326 nm and emits between 350 and 450 nm, making it a suitable FRET partner for tryptophan. [See supporting information for the structure and full chemical name of MQ290.]

**FIGURE 1:**
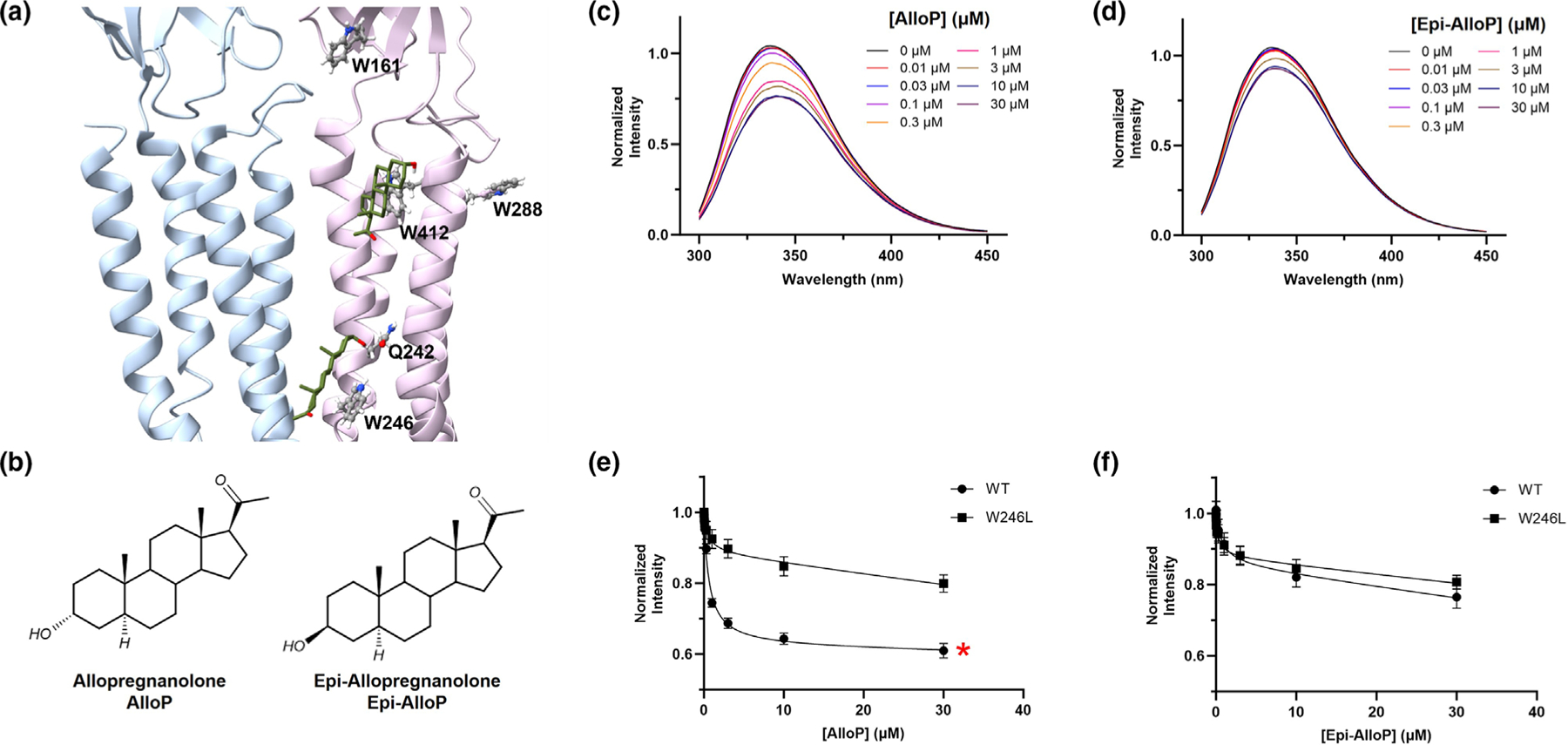
NS quenching of tryptophan residues defines two distinct binding sites in ELIC-α1GABA_A_R. (a) Docking poses of AlloP in the intersubunit and α1-intrasubunit binding sites in the X-ray crystallographic structure of a homo-pentameric ELIC-α1GABA_A_R (PDB: 6CDU). Adjacent subunits are coloured in blue and lavender to emphasize the interface between two identical subunits. The side chains of tryptophan residues (W246, W412, W288 and W161) and glutamine 242 (Q242) are shown as ball and stick drawings. AlloP is shown as a stick drawing in olive with the lowest energy poses in the intersubunit site adjacent to W246 and the α1-intrasubunit site adjacent to W412. (b) Structures of AlloP and Epi-AlloP. The 17-methylketone on the D-ring is responsible for quenching of tryptophan by photo-induced electron transfer. (c, d) Fluorescence emission spectra of ELIC-α1GABA_A_R (0.3 μM) following excitation at 280 nm in the presence of indicated concentrations of AlloP and Epi-AlloP. (e, f) Concentration–dependent quenching of tryptophan emission by AlloP and Epi-AlloP in wild-type receptors and receptors with a W246L mutation. Data are normalized such that peak emission in the absence of NS is set to a value of one. The extent of quenching by AlloP (panel e) is significantly different between WT and W246L; *P < 0.05.

In the current study, we measured NS binding to ELIC-α1GABA_A_R, a pentameric GABA_A_-receptor chimeric protein in which each subunit consists of the TMD of the GABA_A_ receptor α_1_ subunit and the extracellular domain (ECD) of the bacterial pentameric ligand-gated ion channel, ELIC (Q. Chen et al., 2018). Photolabelling and X-ray crystallographic evidence demonstrate that PAM-NS bind at the intersubunit (Q. Chen et al., 2018; Sugasawa et al., 2019) and α_1_-intrasubunit (Sugasawa et al., 2019) sites in ELIC-α1GABA_A_R. Each subunit of ELIC-α1GABA_A_R contains eight tryptophan residues, three of which are in the TMD (W246, W288 and W412) (Figure 1a). PAM-NS bound in the intersubunit site directly interact with W246, and NS docked in the intrasubunit site are proximal to W412 and W288 (Figure 1a). The remaining five tryptophan residues are located in the ELIC-derived ECD domain, three near the N-terminus (W43, W66 and W72) and two in the β8′-β9 loop (W160 and W161). The N-terminal tryptophan residues are >40 Å from the putative intrasubunit site and >70 Å from the intersubunit site. W160 and W161 are 12–14 Å from the putative intrasubunit site and >35 Å from the intersubunit site. ELIC-α1GABA_A_R is readily expressed and purified in large quantities and is stable in purified form, facilitating the development and implementation of steady-state fluorescence-based binding assays. In this study, we measured the binding constants for 3α- and 3β-OH NS to the intersubunit and intrasubunit sites on the GABA_A_ receptor α_1_ subunit. Additionally, inhibition of MQ290 binding enabled us to measure the site-specific binding constants for multiple NS (PAM and NAM) and to examine the effect of the critical Q242L mutation on NS binding to the canonical intersubunit site.

## 2 | METHODS

### 2.1 | Construct design

The ELIC-α1GABA_A_R construct was prepared according to the previously reported procedure (Kinde et al., 2016; Sugasawa et al., 2020). Briefly, the construct was prepared by fusing the ECD of *Erwinia chrysanthemi* ligand-gated ion channel (ELIC) to the TMD of human GABA_A_R-α1. The amino acid sequence of the intracellular loop of GABA_A_R-α1 was substituted by a -GVE- sequence. The N-terminus of the construct contains a deca-histidine tag and a maltose binding protein (MBP). To facilitate MBP cleavage, a tobacco etch virus protease (TEV) protease cleavage site was inserted between MBP and ELIC-α1GABA_A_R.

### 2.2 | Expression and purification

ELIC-α1GABA_A_R was expressed and purified according to a previously published protocol (Q. Chen et al., 2018). The purified protein was aliquoted and stored at −80°C. Mutagenesis was performed as described in previous reports (Kinde et al., 2016) by oligonucleotide-directed mutagenesis using Phu polymerase (Thermo Fisher Scientific, Waltham, MA, USA) verified by sequencing. ELIC-α1GABA_A_R was expressed in OverExpress^™^ C43(D3) *E. coli*, derived from Rosetta^™^(DE3) cells under double selection with kanamycin and chloramphenicol for ELIC-α1GABA_A_R.

### 2.3 | Materials

The synthesis and structural validation of MQ290 and YX03 are described in Supporting Information. The details of ganaxolone (GX) and epi-ganaxolone (Epi-GX) synthesis were reported previously (Hogenkamp et al., 1997). AlloP, PREG, Epi-AlloP and Epi-PREG were purchased from Sigma-Aldrich and Cayman Chemicals. All NS compounds were dissolved in ethanol to a final concentration of 10 mM and stored at −20°C. GABA, propofol and cysteamine were purchased from Sigma-Aldrich.

### 2.4 | General method for fluorescence measurements

Excitation and emission spectra were obtained using a FluoroMax Plus-C fluorometer (Horiba Instruments, Tokyo, Japan). For obtaining emission spectra, 280 nm was used as the excitation wavelength and emission was recorded from 300 to 450 nm. The excitation and emission slit widths used for obtaining emission spectra were 1 and 5 nm. For protein-ligand binding experiments, three emission spectra were obtained and averaged after each addition of a ligand or sodium iodide (NaI). The averaged spectra were smoothed using the Savitzky–Golay method before analysis.

### 2.5 | Photo-physical properties of MQ290

The excitation spectrum of MQ290 (10 μM) was obtained in Buffer A (20-mM Tris pH 7.5, 100-mM NaCl) + 0.015% n-dodecyl β-D-maltoside (DDM). The absorbance spectrum of MQ290 was obtained using a 100-μM solution in ethanol. The extinction coefficients of MQ290 for individual wavelengths were calculated using the Beer–Lambert law. Emission spectra were obtained using 10-μM MQ290 in various solvents. The absorbance spectrum of MQ290 was obtained using a Beckman Coulter DU 530 spectrophotometer.

### 2.6 | AlloP and Epi-AlloP quenching of ELIC-α1GABA_A_R tryptophan fluorescence

One hundred microlitres of purified ELIC-α1GABA_A_R (0.3 μM in Buffer A + 0.015% DDM) was transferred into a 50-μl window quartz cuvette (Starna Cells, Atascadero, CA, USA), which was placed in a cuvette holder maintained at 4°C. The cuvette containing the protein sample remained in the cuvette holder for the duration of the experiment. Protein stability was ensured by comparing the intensities of emission spectra of ELIC-α1GABA_A_R at 330 nm before and after 30 min incubation, and the protein sample was discarded if the intensity decreased by more than 1%. Two microlitres of NaI (AK Scientific, Union City, CA, USA; 5-M stock prepared in 0.1-Mm sodium hydrosulfite [Sigma-Aldrich, St. Louis, MO, USA]) was added to the protein (final concentration = 100 mM) and incubated for 10 min to reduce the fluorescence emission from hydrophilic tryptophans. To measure the concentration–dependent effects of NS on tryptophan fluorescence, 1 μl of NS from each 100× stock solution was sequentially added to the NaI-equilibrated protein and thoroughly mixed to achieve concentrations from 0.01 to 50 μM. The protein-NS solution was incubated for 30 min after each addition. Following each incubation period, the emission spectrum of the protein-NS solution was acquired. The binding of AlloP and Epi-AlloP to ELIC-α1GABA_A_R was monitored by measuring tryptophan quenching as determined by the intensity of emission of ELIC-α1GABA_A_R at 330 nm.

### 2.7 | Binding of MQ290 to ELIC-α1GABA_A_R

To monitor binding of MQ290 to ELIC-α1GABA_A_R, 100 μl of ELIC-α1GABA_A_R (0.3 μM) was transferred into a 50-μl window quartz cuvette that was placed in a cuvette holder. The temperature of the cuvette was maintained at 4°C. NaI (final concentration = 100 mM) was added to the protein sample and incubated for 10 min to reduce the emission of hydrophilic tryptophan residues. One-microlitre aliquots of 100× stock solutions of MQ290 (prepared in Buffer A + 0.015% DDM) were sequentially added to the protein sample and incubated for 30 min after each addition. Because MQ290 alone absorbs at 280 nm, emission spectrum (background spectrum) of MQ290 in the absence of protein was also obtained. The MQ290 background spectra were subtracted from the protein-MQ290 emission spectra to obtain the background-corrected spectra and were smoothened using the Savitzky–Golay method before analysis.

### 2.8 | Estimation of MQ290 emission spectrum as a result of tryptophan-MQ290 FRET

The ELIC-α1GABA_A_R emission spectrum obtained before the addition of MQ290 was used as the reference to estimate the contribution of ELIC-α1GABA_A_R tryptophan emission in the presence of MQ290. Before addition of MQ290, the emission intensities of ELIC-α1GABA_A_R from 300 to 450 nm were divided by the intensity of emission at 330 nm. These ratios were unchanged by the addition of AlloP to ELIC-α1GABA_A_R, indicating that NS addition does not shift or change the shape of the ELIC-α1GABA_A_R emission spectrum. Therefore, we multiplied these ratios by the intensity of the spectra of ELIC-α1GABA_A_R plus MQ290 at 330 nm to calculate the contribution of tryptophan emission to the MQ290 plus ELIC-α1GABA_A_R spectra at each wave-length. We selected the 330-nm intensity to estimate the tryptophan emission of ELIC-α1GABA_A_R, because MQ290 has minimal emission at 330 nm. The calculated ELIC-α1GABA_A_R tryptophan emission spectrum was then subtracted from the background-corrected spectrum to obtain the emission spectrum of the signal resulting from energy transfer (FRET spectrum) from tryptophan to MQ290 (Figure S6). The 370-nm intensity values of the FRET spectra were plotted against MQ290 concentration to yield the MQ290 binding curve.

### 2.9 | Inhibition curves of 3α-OH and 3β-OH NS

One hundred microlitres of ELIC-α1GABA_A_R (0.3 μM) was transferred into a 50-μl window quartz cuvette maintained at 4°C, and NaI was added to the protein sample to a final concentration of 100 mM and incubated for 10 min. MQ290 was then added at a concentration of 3 μM for 3α-OH NS inhibition curves or 1 μM for 3β-OH NS inhibition curves and incubated for 30 min. One-microlitre aliquots of 3α-OH and 3β-OH NS from their corresponding stock solutions (prepared in Buffer A + 0.015% DDM) were added to achieve concentrations between 0.01 and 30 μM. The background emission spectra of ligands alone were obtained for each ligand concentration in the absence of protein and were subtracted from the protein-ligand emission spectra to yield background subtracted data. The MQ290 FRET spectra for different ligand concentrations were estimated using the method described above. The 370-nm intensities of the FRET spectra were plotted against ligand concentration and fitted to a four-parameter logistic curve to obtain ${\mathrm{IC}}_{50}$ data (GraphPad Software version 9.4.0, San Diego, CA). $K_{i}$ values were calculated using the relationship:

$$K_{i}\;=\;\left[IC_{50}\right]/\left(1\;+\;\left[L_{T}\right]/K_{d}\right)$$

where $L_{T}$ is the concentration of MQ290 and $K_{d}$ was determined from the MQ290 binding curve.

### 2.10. | Photolabelling and middle-down MS analysis

The synthesis of the NS photolabelling reagent KK200 is detailed in previous reports (W. W. L. Cheng et al., 2018; Jiang et al., 2016). For the photolabelling competition experiments, 20 μg of ELIC-α1GABA_A_R was incubated with 3-μM KK200 in the presence of 30-μM competitor (AlloP, Epi-AlloP or MQ290) or the same volume of ethanol at 4°C for 1 h. The samples were then irradiated in a quartz cuvette for 5 min, using a photoreactor emitting light at >320 nm (Germann et al., 2021). Sample preparation for tryptic digestion and analysis by LC–MS was performed as previously described (Sugasawa et al., 2019). Peptides were separated on a home-packed PLRP-S (Agilent, Santa Clara, CA, USA) column (10 cm × 75 μm, 300 Å) using a 135-min gradient from 10% to 90% acetonitrile and introduced to an Orbitrap-Elite mass spectrometer (Thermo Fisher Scientific, Waltham, MA, USA; RRID:SCR_020548) at 800 nl·min^−1^ with a nanospray source. MS acquisition was set as an MS1 Orbitrap scan (resolution of 60,000) followed by top 20 MS2 Orbitrap scans (resolution of 15,000) using data-dependent acquisition and exclusion of singly charged precursors. Fragmentation was performed using high-energy dissociation with normalized energy of 35%. Analysis of data sets was performed using Xcalibur (Thermo Fisher Scientific, Waltham, MA, USA; RRID: SCR_014593) to manually search for TM1, TM2, TM3 or TM4 tryptic peptides with or without NS photolabelling modifications. Photolabelling efficiency was estimated by generating extracted chromatograms of unlabelled and labelled peptides, determining the area under the curve, and calculating the (area of labelled peptide/(area of unlabelled + area of labelled peptide). MS2 spectra of photolabelled TMD peptides were analysed by manual assignment of fragment ions with and without photolabelling modification.

### 2.11 | Receptor expression and electrophysiological recordings

Receptor expression and electrophysiological recordings were conducted as described in detail previously (Sugasawa et al., 2020). In brief, α_1_β_3_ GABA_A_ receptors were expressed in oocytes from the African clawed frog (*X. laevis*). The wild-type α_1_ and β_3_ subunits were of human origin (Genbank accession numbers NM_000806.5 and NM_000814.5, respectively). The α_1_ clone containing the Q-to-L mutation to the classic neurosteroid binding site (Hosie et al., 2006) was of rat origin. (The glutamine residue in ELIC-α1GABA_A_R is Q242, whereas the corresponding residue in rat α_1_ is Q241. For simplicity, the residue is referred to as Q242 for both rat and ELIC-α1GABA_A_R.) The oocytes were purchased as quarter ovaries from Xenopus1 (Dexter, MI, USA). Following digestion in 2% w/v (mg·ml^−1^) collagenase A, the oocytes were injected with a total of 12 ng cRNA per oocyte in a 5:1 ratio (α1:β3) and incubated at 15°C for 2–3 days prior to electrophysiological recordings in ND96 (96-mM NaCl, 2-mM KCl, 1.8-mM CaCl_2_, 1-mM MgCl_2_, 5-mM HEPES; pH 7.4) supplemented with 100 U·ml^−1^ penicillin and 100 μg·ml^−1^ streptomycin.

Electrophysiological recordings were conducted at ≈22°C using standard two-electrode voltage clamp. The voltage and current electrodes (G120F-4, OD = 1.20 mm, ID = 0.69 mm; Warner Instruments, Hamden, CT, USA) were filled with 3-M KCl and had resistances of 0.3–1 MΩ. The oocytes were clamped at −60 mV. The chamber (RC-1Z; Warner Instruments) was perfused with ND96 at 5–8 ml·min^−1^. Solutions were gravity-applied. The current responses were amplified with an Axoclamp 900A (Molecular Devices, San Jose, CA, USA) or OC-725C amplifier (Warner Instruments), digitized with a Digidata 1200 series digitizer (Molecular Devices) and stored on PC hard drive using pClamp (Molecular Devices; RRID:SCR_011323). Current amplitudes were determined using Clampfit (Molecular Devices).

A standard experiment consisted of a several-minute long applications of GABA followed immediately by a co-application of GABA + modulator and a wash in GABA. For normalization purposes, each cell was exposed to the combination of saturating (1-mM) GABA + 50-μM propofol, assumed to fully activate all receptors in the cell (Eaton et al., 2016).

The stock solution of GABA was made in ND96 bath solution at 500 mM and stored in aliquots at −20°C. The steroids were dissolved in DMSO at 10–20 mM and stored at room temperature. Dilutions to working concentrations were made on the day of experiment. The maximal final concentration of DMSO was 0.15%. In control experiments on the α_1_β_3_ receptor, 0.1% DMSO did not significantly modify the steady-state response to 0.5-μM GABA (probability of being in the active state, *P*_*A*_ = 0.03 ± 0.01; n = 5 cells) or to 1-mM GABA (a saturating concentration, *P*_*A*_ = 0.70 ± 0.08; n = 5). The application of 0.5% DMSO reduced the response to 0.5-μM GABA (*P*_*A*_ = 0.05 ± 0.01; n = 5) to 86 ± 6% of control but was without effect on the response to 1-mM GABA (*P*_*A*_ = 0.81 ± 0.23; n = 5).

### 2.12 | Molecular docking

The molecular coordinates of AlloP (PubChem CID: 92786) and epi-AlloP (PubChem CID: 93787) were obtained from PubChem. The docking template of ELIC-α1GABA_A_R was generated using the X-ray crystallographic structure (PDB:6CDU). The ligands bound to the protein were deleted and the structure was energy minimized in Chimera ver. 1.16 (Pettersen et al., 2004; RRID:SCR_004097). DockPrep was used to add hydrogens and charges to the protein structure. Grids of the following sizes were generated to encompass the putative neurosteroid binding sites: intersubunit site (14 × 12 × 15 Å); intrasubunit site near W412 (16 × 16 × 15 Å); intrasubunit site near W288 (15 × 15 × 18 Å). Docking was performed using AutoDock Vina (Trott & Olson, 2010) in the Chimera software to obtain the preferred binding poses of AlloP and epi-AlloP shown in Figure 1a.

### 2.13 | Calculation of first singlet excited state energies of MQ290 and tryptophan

For a direct electron transfer mechanism, the energy of the first singlet excited state of the donor molecule should match with the energy of the singlet excited state of the acceptor molecule. These energies were obtained computationally using density function theory. The initial guess geometries of MQ290 and tryptophan were generated using Avogadro software (RRID:SCR_015983). The structures were optimized and energies of the first singlet excited states of MQ290 and tryptophan were calculated using B3LYP/def2-TZVP def2/J RIJCOSX TIGHTSCF D3BJ level of theory. The geometry optimization and energy calculations were performed using ORCA version 5.0.4 software (Neese, 2012).

### 2.14 | Distance estimations

The Förster distance $\left(R_{0}\right)$ was estimated using the absorption spectrum of MQ290, emission spectrum of ELIC-α1GABA_A_R and reported fluorescence quantum yield of tryptophan (Tine & Aaron, 1984). The following equation was used for $R_{0}$ estimation.


$$R_{0}\;=\;0.211{\left(\kappa^{2}\eta^{-4}QJ\left(\lambda \right)\right)}^{1/6}\;\left(\mathrm{in}\;Å\right),$$

where $\kappa^{2}$ is the orientation factor, Q is the fluorescence quantum yield of tryptophan (0.07) and $J\left(\lambda \right)$ is the overlap integral. A $\kappa^{2}$ value of 2/3, the value for two randomly oriented molecules, was used in the calculation because the precise value of donor and acceptor in the ligand bound position is not known. This provides an estimate rather than a precise measurement of $R_{0}$. A value of η=1.4 (the refractive index of water) was used since water was the experimental solvent.

The distance between bound MQ290 and tryptophan in the intersubunit binding site was estimated using the following equation.


$$1\;-\;\frac{F_{DA}}{F_{D}}\;=\;\frac{1}{1\;+\;{\left(\frac{r}{R_{0}}\right)}^{\frac{1}{6}}}$$

where r is the distance between donor and acceptor, $R_{0}$ is the Förster distance, $F_{D}$ is the emission intensity of W246 in the absence of MQ290 and $F_{DA}$ is the emission intensity of W246 in presence of MQ290. The difference between the emission intensities of ELIC-α1GABA_A_R WT and ELIC-α1^W246L^GABA_A_R at 330 nm was considered as the emission intensity of W246. The change in the emission intensity of ELIC-α1GABA_A_R at 330 nm due to specific binding of 10-μM MQ290 was considered as F_DA_.

### 2.15 | Statistical analysis

All statistical analyses were performed with GraphPad Prism (version 9.4.0, GraphPad Software, San Diego, CA; RRID:SCR_002798). For MQ290 binding assay and competition experiments, the curve-fitting program was used to fit concentration-effect relationships to a four-parameter logistic equation. Analysis of statistical significance comparing the photolabelling efficiency of KK200 was determined using one-way ANOVA with Dunnett’s multiple comparisons test. Analysis of statistical significance comparing the current response to GABA vs. (GABA + NS) was performed using paired two-way *t*-test. Statistical significance of the difference in a single binding parameter (e.g., maximal response, $K_{d}$, $K_{i}$, etc.) between two conditions (e.g., wild type vs. a mutation) was analysed using unpaired two-way *t*-tests. Comparison of three or more variables was performed using a one-way ANOVA followed by a Tukey’s multiple comparisons test. Post hoc tests were conducted only if F was significant, and there was no variance inhomogeneity. A *P* value of <0.05 was considered as significant and * indicates *P* < 0.05. The data and statistical analysis comply with the recommendations of the *British Journal of Pharmacology* on experimental design and analysis in pharmacology.

### 2.16 | Nomenclature of targets and ligands

Key protein targets and ligands in this article are hyperlinked to corresponding entries in http://www.guidetopharmacology.org, and are permanently archived in the Concise Guide to Pharmacology 2021/22 (Alexander et al., 2021).

## 3 | RESULTS

### 3.1 | NS quenching of specific tryptophans on ELIC-α1GABA_A_R

To determine whether NS binding can be measured by proximity to tryptophan in its binding pockets, we examined the ability of AlloP (Figure 1b) to quench the emission of ELIC-α1GABA_A_R (peak emission ~335 nm) elicited by excitation at 280 nm. AlloP produced concentration–dependent quenching that saturated at concentrations above 3 μM (Figure 1c,e). The same pattern of quenching was observed with excitation at 295 nm, indicating that AlloP quenches tryptophan rather than tyrosine residues. AlloP quenching was eliminated by denaturation of the protein in 2% SDS indicating that quenching is dependent on the conformational integrity of the NS binding sites (Figure S1). The likely mechanism of quenching is photo-induced electron transfer (PET) between the excited state of a tryptophan in a NS binding pocket and the 17-methylketone group of AlloP. To confirm this, we examined the ability of YX03 (Figure S2), a NS analogue in which the 17-methylketone is replaced by a methoxy group, to quench tryptophan fluorescence. YX03 potentiates GABA-elicited currents in α_1_β_3_ GABA_A_ receptors, and its effect is blocked by the α_1_-Q242L mutation, indicating that YX03 binds to the canonical intersubunit NS binding site. YX03 does not quench ELIC-α1GABA_A_R tryptophan fluorescence, consistent with the absence of the 17-methylketone responsible for quenching. YX03 also partially prevents AlloP induced quenching (Figure S2e). This is consistent with YX03 competing with AlloP binding at the intersubunit NS binding site, but perhaps not in the α_1_-intrasubunit binding site (see below).

PET occurs over very short (<5 Å) distances, indicating that NS binding should produce quenching of specific tryptophan residues. AlloP binds in the canonical intersubunit binding site where it has a ring stacking interaction with W246 and in the α_1_-intrasubunit binding site where it may quench one or more of the tryptophan residues surrounding the binding pocket (W412 or W288, and possibly W161) (Figure 1a). Epi-AlloP, in contrast, binds only to the α_1_-intrasubunit site and should not quench W246. Epi-AlloP produces concentration-dependent quenching of ELIC-α1GABA_A_R fluorescence (saturating at concentrations above 10 μM) but to a lesser extent than AlloP (Figure 1d,f). The combination of saturating concentrations of AlloP and Epi-AlloP quench tryptophan fluorescence to the same extent as does a saturating concentration of AlloP alone, regardless of the order of addition (Figure S3). This indicates that the Epi-AlloP binding site is a subset of the AlloP binding sites, consistent with AlloP binding in both the intersubunit and intrasubunit sites, while Epi-AlloP binds only in the intrasubunit site.

To distinguish between AlloP binding in the intersubunit and intrasubunit binding sites, AlloP quenching was compared between WT receptors and receptors with a W246L mutation (intersubunit site). AlloP quenching is reduced 66 ± 6% (*P* < 0.05) in the W246L receptors, indicating that binding in the intersubunit site is the major contributor to quenching. While the stoichiometry of AlloP binding in the intersubunit and intrasubunit binding sites is presumably 1:1, W246 in the intersubunit site likely contributes more of the observed quenching because the 17-methylketone is closer to W246 than to the tryptophan residue(s) in the intrasubunit site. The observed AlloP ${\mathrm{IC}}_{50}$ value of 0.64 ± 0.07 μM in WT receptors represents a weighted average of the $K_{d}$ values for binding to the intersubunit and intrasubunit sites. There was residual (29 ± 1%), saturable quenching in the W246L receptors with an ${\mathrm{IC}}_{50}$ value of 0.50 ± 0.21 μM, representing the ${\mathrm{IC}}_{50}\left(K_{d}\right)$ for AlloP binding in the intrasubunit site (Figure 1e). Epi-AlloP quenches tryptophan fluorescence in WT and W246 receptors to a similar extent indicating that Epi-AlloP does not quench W246 and binds only in the intrasubunit site (WT ${\mathrm{IC}}_{50}\;=\;0.71\;\pm \;0.32$; W246L ${\mathrm{IC}}_{50}\;=\;0.34\;\pm \;0.13$; N.S. *P* = 0.57) (Figure 1f). The extent of Epi-AlloP quenching was similar to the extent of AlloP quenching observed in W246L receptors, consistent with AlloP and Epi-AlloP binding in the intrasubunit site. The $K_{d}$ of AlloP binding to the intersubunit site was determined by measuring AlloP quenching in the presence of a saturating (30 μM) concentration of Epi-AlloP. The $K_{d}$ value was 0.26 ± 0.04 μM, indicating that AlloP binds with similar affinity to the intersubunit and intrasubunit sites.

### 3.2 | Synthesis and characterization of MQ290

**FIGURE 2:**
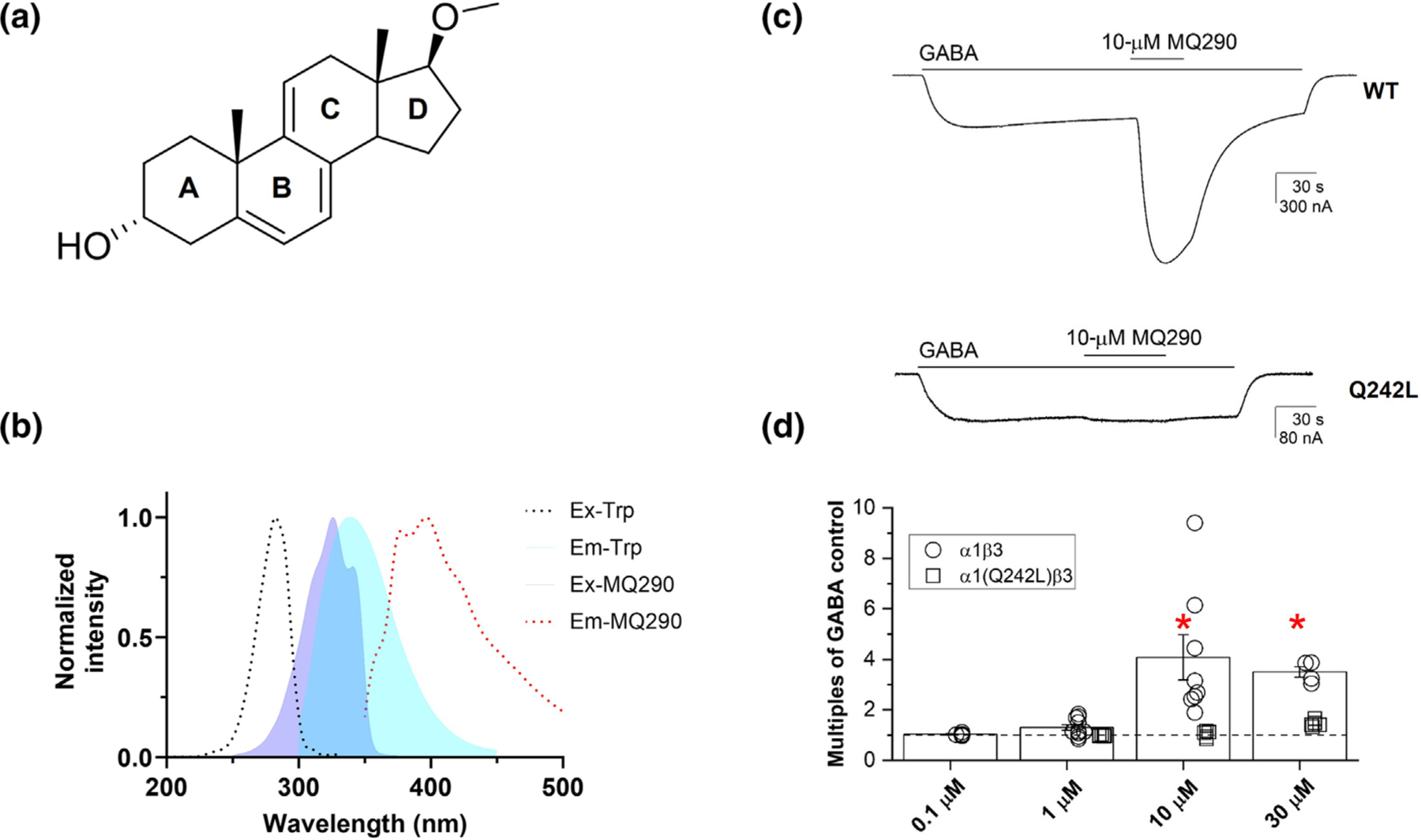
Spectral and functional properties of MQ290. (a) Structure of MQ290. (b) Fluorescence excitation and emission spectra for ELIC-α1GABA_A_R tryptophan and MQ290. The overlap of tryptophan emission (violet) and MQ290 excitation (cyan) is shown in blue. (c and d) Effects of MQ290 on GABA-elicited currents in α_1_β_3_ GABA_A_ receptors expressed in *Xenopus* oocytes. (c) Representative traces of currents elicited by low concentrations of GABA (0.02–0.03 μM; P_A_ = 0.03–0.10 for WT and 0.3–1.0 μM; P_A_ = 0.08 for Q241L) in α_1_β_3_ (top) and α_1_^Q242L^β_3_ (bottom) GABA_A_ receptors. (d) Enhancement of currents elicited with low GABA (n = 3–8) by MQ290 (0.1–30 μM) in α_1_β_3_ (○: open circles) and α_1_^Q241L^β_3_ (□: open squares). The y-axis shows the ratio of the response of GABA + MQ290 to GABA alone, with a value of 1 indicating no MQ290 enhancement. * indicates that in α_1_β_3_, but not α_1_^Q242L^β_3_ receptors, 10- and 30-μM MQ290 enhance (*P* < 0.05) the currents elicited by GABA.

To develop a PAM NS that could serve as a FRET acceptor for a localized tryptophan donor, we sought to design a molecule that (1) does not chemically quench tryptophan, (2) has the appropriate excitation and emission spectra (ex_max_ ~ 330 nm and em_max_ > 350 nm) and (3) retains the GABA_A_ receptor PAM activity of NS. Accordingly, we synthesized MQ290, a NS analogue with a 17-methoxy substituent, a 5–6, 7–8, 9–11 triene ring structure and a 3α-OH group (Figure 2a). The three conjugated double bonds in the B and C rings of the NS were introduced to mimic the properties of fluorescent analogues of cholesterol (Liu et al., 2009) and ergosterol (K. H. Cheng et al., 1999). The details of synthesis and structural characterization are provided in Supporting Information. The excitation and absorption spectra of MQ290 have maxima of 326 nm and substantially overlap with the tryptophan emission spectrum (Figure 2b), a condition necessary for resonance energy transfer. The emission maximum of MQ290 is 396 nm in aqueous solution (Buffer A and 0.015% DDM) (Figure 2b) but varies with solvent environment; the emission maxima shifts to lower wavelengths (blue shift) as solvent polarity decreases with an emission maximum of 370 nm in hexanes (Figure S4). The peak emission at 370 nm observed with hexanes represents the maximum difference between the expected emission of MQ290 in a hydrophobic binding pocket and an aqueous environment. MQ290 also retains the characteristic functional effects of PAM neurosteroids. It potentiates GABA-elicited currents in α_1_β_3_ GABA_A_ receptors with an EC_50_ value between 1 and 10 μM and the potentiating effect is eliminated by the α_1_(Q242L) mutation (Figure 2c,d). Based on these data we conclude that MQ290 is a PAM-NS with appropriate spectral properties to function as a FRET acceptor for tryptophan.

### 3.3 | MQ290 prevents photolabelling of the intersubunit site

To confirm that MQ290 binds in the intersubunit site, we examined the ability of MQ290 to prevent photolabelling of the intersubunit binding site in ELIC-α1GABA_A_R. The NS analogue photolabelling reagent, KK200, labels the intersubunit site at the Y309 residue on TM3 in ELIC-α1GABA_A_R (Sugasawa et al., 2019). We examined whether a 10-fold excess of MQ290, AlloP (positive control) or Epi-AlloP (negative control) prevents KK200 (3 μM) photolabelling of the TM3 peptide. MQ290 and AlloP prevented KK200 labelling of TM3 whereas Epi-AlloP did not (Figure S5), consistent with MQ290 binding to the intersubunit site.

### 3.4 | Binding of MQ290 to ELIC-α1GABA_A_R produces a saturable FRET signal

**FIGURE 3:**
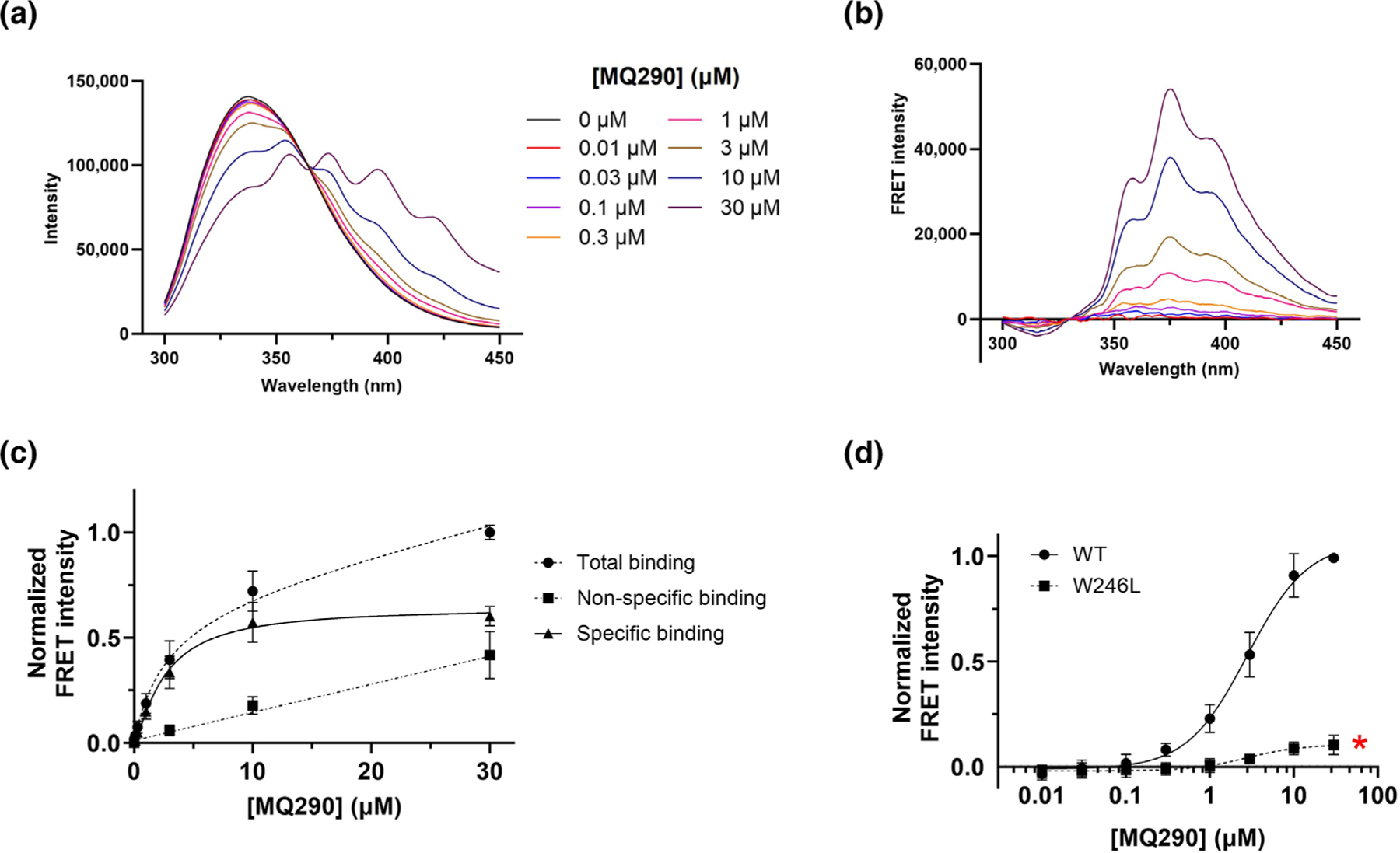
Tryptophan–MQ290 FRET signal reflects a specific interaction with W246 in the intersubunit binding pocket in ELIC-α_1_GABA_A_R. (a) Fluorescence emission spectra (Ex = 280 nm) of ELICα_1_-GABA_A_R (0.3 μM) in the presence of varying concentrations of MQ290 (0–30 μM). (b) Extracted tryptophan-MQ290 FRET signal from spectra in panel a; the FRET signal is isolated by subtraction of the emission spectra of MQ290 without protein and of the contribution of tryptophan emission to the spectra (see Figure S6). (c) FRET intensity as a function of MQ290 concentration. The figure plots peak intensity of the FRET spectra (370 nm) in the absence (●: total) or presence (■: non-specific) of 30-μM AlloP, where AlloP is a competitive inhibitor of MQ290 binding (see Figure 4a). Subtraction of the non-specific signal from total signal yields the specific FRET binding signal (▲) with a $K_{d}\;=\;2.60\;\pm \;0.35\;\text{μM}$. All values are normalized to the total FRET signal at 30 μM with n = 5 for each data point. (d) Specific FRET signal plotted as a function of MQ290 concentration in wild-type (WT) ELIC-α1GABA_A_R and ELIC-α_1_^W246L^GABA_A_R (n = 5 ± SD for each data point). * indicates that the maximal FRET signal is different (*P* < 0.05) between WT and W246L receptors.

**FIGURE 4:**
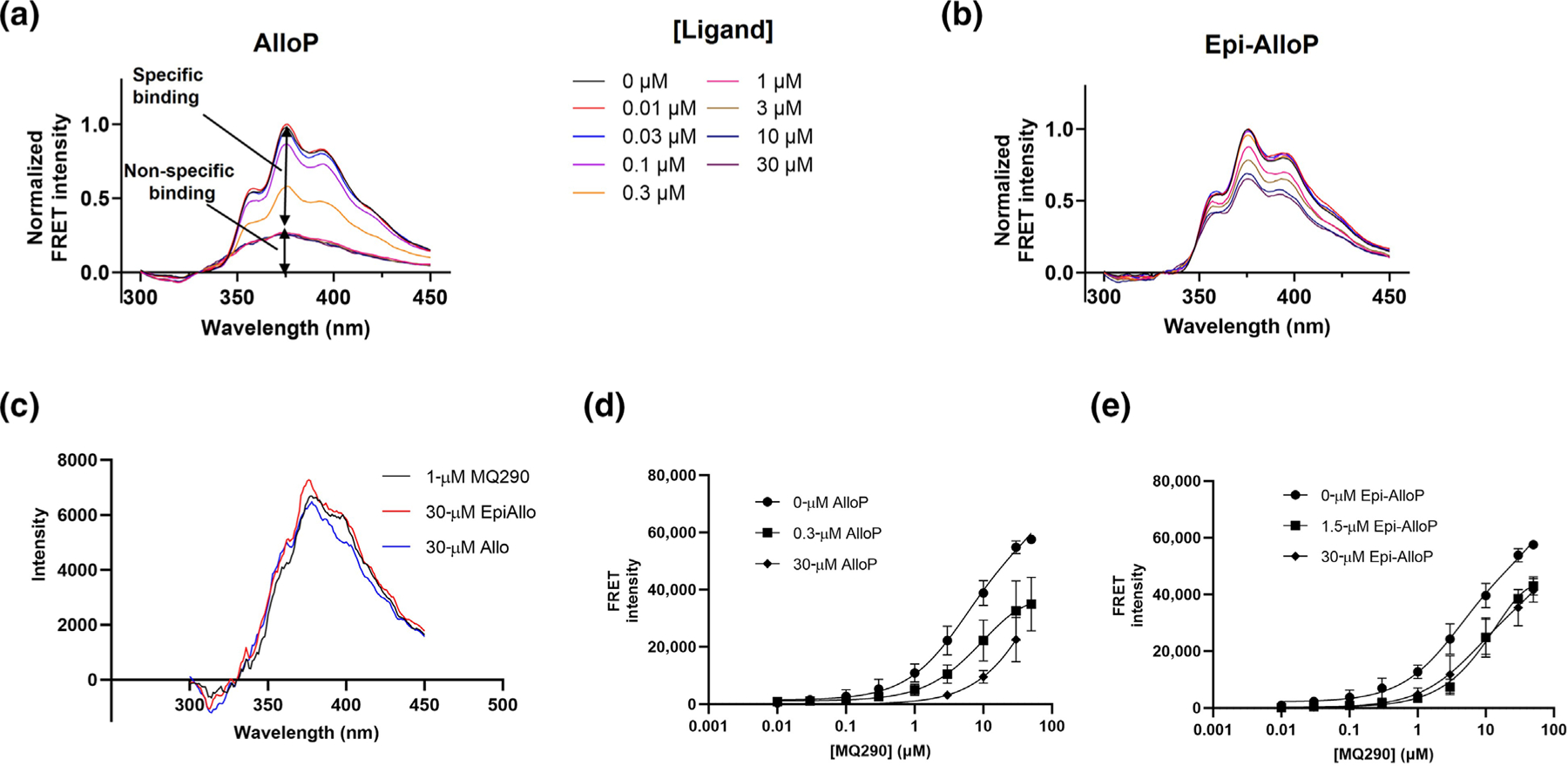
AlloP and Epi-AlloP inhibit FRET between ELIC-α1GABA_A_R and MQ290. (a) Extracted FRET spectra of MQ290 (3 μM) in the absence or presence of AlloP (0.01–30 μM). Inhibition of the FRET signal saturates at AlloP concentrations ≥ 1 μM. (b) Extracted FRET spectra of MQ290 (1 μM) in the absence or presence of Epi-AlloP (0.01–30 μM). Inhibition of the FRET signal saturates at Epi-AlloP concentrations ≥ 10 μM. (c) Extracted FRET spectra of MQ290 (1 μM) with ELIC-α_1_^Q246L^GABA_A_R in the absence or presence of 30-μM concentrations of AlloP and Epi-AlloP. (d) Concentration-dependence of extracted MQ290-ELIC-α1GABA_A_R FRET in the presence of 0.3-μM $\left(3\;\times \;K_{d}\right)$ and 30-μM AlloP (n = 5 ± SD at each point). (e) Concentration-dependence of extracted MQ290-ELIC-α1GABA_A_R FRET in the presence of 1.5-μM $\left(3\;\times \;K_{d}\right)$ and 30-μM Epi-AlloP (n = 5 ± SD at each point).

To determine if MQ290 binding to ELIC-α1GABA_A_R produces a specific FRET signal, we analysed the concentration-dependent effects of MQ290 on the ELIC-α1GABA_A_R tryptophan emission spectrum. MQ290 produced concentration-dependent reduction of tryptophan emission at 330 nm and enhancement of MQ290 emission at 370 nm, consistent with a FRET interaction (Figure 3a). To isolate the FRET signal we subtracted both the emission spectra of MQ290 in the absence of ELIC-α1GABA_A_R protein and the direct contribution of tryptophan emission to the spectra for each concentration of MQ290 (Figure S6). Tracings of the isolated FRET signal as a function of MQ290 concentration are shown in Figure 3b. The MQ290 FRET signal was markedly reduced in the presence of an excess of a NS competitor (30-μM AlloP; Figure 4a); a small non-specific FRET signal remained and specific FRET (a surrogate for specific binding) was calculated by subtraction of the nonspecific FRET signal from the total FRET signal. Total, non-specific and specific FRET signal are plotted as a function of MQ290 concentration in Figure 3c. The $K_{d}$ for specific MQ290 binding was 2.60 ± 0.35 μM. The MQ290 $K_{d}$ value was not significantly changed $\left(K_{d}\;=\;1.94\;\pm \;0.72\;\text{μM}\right)$ in the presence of a saturating concentration (10 mM) of the orthosteric agonist cysteamine. To determine the relative contribution of the two NS binding sites to the FRET signal, we compared specific MQ290 FRET between WT and W246L ELIC-α1GABA_A_Rs. The maximal MQ290 FRET signal is reduced ~90% (*P* < 0.001) in W246L receptors (Figure 3d), indicating that W246 in the intersubunit site contributes almost all the FRET signal.

### 3.5 | Competitive inhibition of MQ290 binding by 3α-OH and 3β-OH NS

**FIGURE 5:**
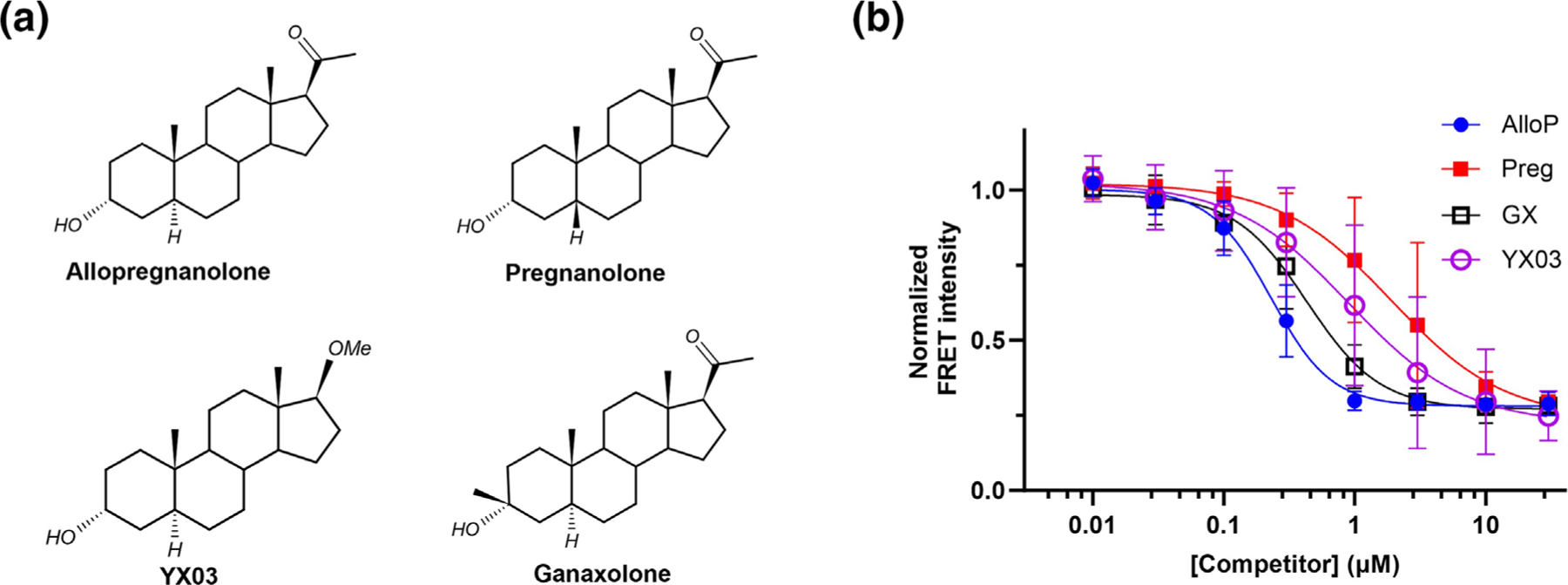
Inhibition of ELIC-α1GABA_A_R–MQ290 FRET by 3α-OH NS. (a) Structures of 3α-OH NS. (b) Inhibition of MQ290 (3 μM) FRET signal. FRET intensity for each sample was normalized to the maximum signal (370 nm) in the absence of competitor (n = 5 ± SE for each data point). ${\text{IC}}_{\text{50}}\;\pm \;\mathrm{SE}$ values for the NS are AlloP = 0.23 ± 0.02 μM; GX = 0.43 ± 0.05; PREG = 1.92 ± 0.64; and YX03 = 0.91 ± 0.35.

To determine the binding affinity of 3α-OH (PAM) and 3β-OH (NAM) NS, we incubated AlloP and Epi-AlloP with ELIC-α1GABA_A_R and measured the concentration-dependent (0.01–30 μM) inhibition of the MQ290 FRET signal. AlloP inhibited the FRET signal by 71 ± 0.02% with an ${\text{IC}}_{\text{50}}$ value of 0.23 ± 0.02 μM (Figures 4a and 5b) and Epi-AlloP inhibited the FRET signal by a lesser extent, with an ${\text{IC}}_{\text{50}}$ value of 0.36 ± 0.04 μM (Figures 4b,e and S6b). To determine if the intrasubunit site contributed to the MQ290 FRET signal, we examined the ability of saturating concentrations (30 μM) of AlloP and Epi-AlloP to inhibit MQ290 FRET in W246L receptors. The MQ290 FRET signal was reduced 90% in W246L (Figure 3d); the remaining FRET signal was reduced by neither AlloP nor Epi-AlloP (Figure 4c). These data indicate that MQ290 directly reports NS binding in the intersubunit site by forming a FRET pair with W246. Since Epi-AlloP inhibition of MQ290 FRET is prevented by the W246L mutation, we reasoned that Epi-AlloP binding to the intrasubunit site allosterically inhibits the MQ290-W246 FRET signal, thus indirectly reporting on 3β-OH NS binding. To confirm that AlloP competitively inhibits whereas Epi-AlloP allosterically modulates MQ290 binding, we measured MQ290 binding curves in the presence and absence of AlloP or Epi-AlloP. AlloP produced a concentration-dependent rightward shift in MQ290 FRET, consistent with a competitive interaction (Figure 4d). In contrast, while Epi-AlloP (at a concentration $3\;\times \;K_{d}$) produced a rightward shift in the MQ290 concentration curve, no further change in $K_{d}$ was observed at a concentration of 30 μM (Figure 4e). These data are not consistent with competition and support an allosteric mechanism of inhibition.

### 3.6 | 3α-OH (PAM) NS

Four 3α-OH NS were evaluated and shown to inhibit MQ290 binding to a similar extent (Figure 5b), consistent with competition for binding at a common (intersubunit) site. PREG had an ${\mathrm{IC}}_{50}$ value three to five times greater than AlloP or GX (Figure 5b and Table 1a), indicating that a *cis* A,B-ring fusion (pregnanolone) reduces NS affinity for the intersubunit binding site. $K_{i}$ values for each NS were calculated using the Cheng-Prusoff relationship (assuming an MQ290 $K_{d}$ value of 2.60 μM) and are reported in Table 1a.

### 3.7 | 3β-OH (NAM) NS

**FIGURE 6:**
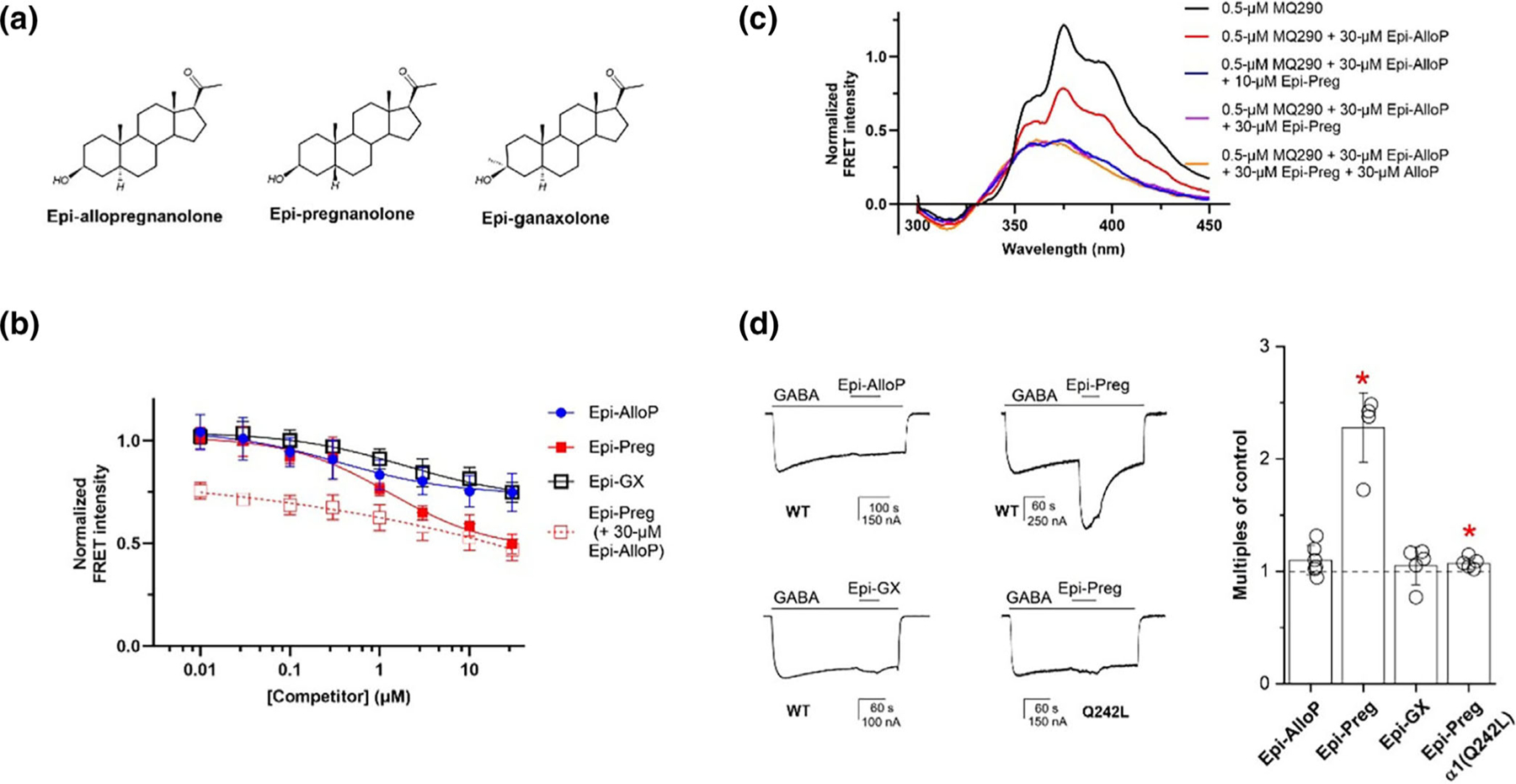
Inhibition of ELIC-α1GABA_A_R–MQ290 FRET by 3β-OH NS. (a) Structures of 3β-OH NS. (b) Inhibition of MQ290 (1 μM) FRET signal. FRET intensity for each sample was normalized to the maximum signal (370 nm) in the absence of competitor (n = 5 ± SD for each data point). ${\text{IC}}_{\text{50}}\;\pm \;\mathrm{SE}$ values for the NS are Epi-AlloP = 0.36 ± 0.30 μM; Epi-GX = 1.92 ± 0.40 μM; Epi-PREG = 1.31 ± 0.42 μM. (c) Inhibition of MQ290 (0.5 μM) FRET signal in ELIC-α1GABA_A_R. The figure shows the extracted FRET spectra of MQ290 in ELIC-α1GABA_A_R in the presence of various combinations of Epi-PREG, Epi-AlloP and AlloP. Epi-PREG adds to the inhibition of the FRET signal produced by a saturating concentration of Epi-AlloP (30 μM) but does not add to the inhibition produced by saturating concentrations of either AlloP (30 μM) or AlloP + Epi-AlloP. (d) Epi-PREG potentiates GABA-elicited currents in α_1_β_3_ GABA_A_ receptors expressed in Xenopus oocytes. (Left) Representative traces showing that Epi-PREG, but not Epi-AlloP or Epi-GX (all 10 μM) potentiates the currents elicited by GABA (0.02–0.03 μM; P_A_ = 0.03–0.10). Epi-PREG potentiation is absent in α_1_^Q242L^β_3_ GABA_A_ receptors ([GABA] = 0.3– 1.0 μM; P_A_ = 0.08), indicating that its action is mediated by the intersubunit binding site. (Right) Enhancement of currents elicited with low GABA (n = 3–8) by 3β-OH NS (10 μM) in wild-type α_1_β_3_ and α_1_^Q242L^β_3_ GABA_A_ receptors. The y-axis shows the ratio of the response to GABA + NS to GABA alone, with a value of 1 indicating no enhancement. * indicates a significant difference (*P* < 0.05) between (NS + GABA) and GABA alone.

We then examined the ability of 3β-OH (NAM) NS to inhibit MQ290 (1 μM) binding to ELIC-α1GABA_A_R. Epi-AlloP and Epi-GX (Figure 6a) reduced the FRET signal to an equal extent (Figure 6b) with $K_{i}$ values five to six times higher than the corresponding values for 3α-OH steroids (Table 1a) (n.b. since the 3β-OH NS inhibit MQ290 FRET allosterically, ${\mathrm{IC}}_{50}\;=\;K_{i}$). In contrast, Epi-pregnanolone (Epi-PREG) inhibited the FRET signal to a greater extent than the other 3β-OH NS (*P* < 0.01). In the presence of a saturating concentration of Epi-AlloP, Epi-PREG further inhibits the FRET signal, suggesting it may also bind to the intersubunit site (Figure 6b). To assess whether Epi-PREG binds to the intersubunit site, we examined inhibition of the MQ290 FRET signal by combinations of Epi-AlloP, Epi-PREG and AlloP, using a low (0.5 μM) concentration of MQ290 to enable complete competition by Epi-PREG (Figure 6c). Epi-PREG added to the inhibition of MQ290 FRET produced by a saturating concentration of Epi-AlloP, with maximal inhibition at 10 μM. No additional inhibition of the FRET signal was produced by 30-μM AlloP, consistent with Epi-PREG occupancy of the intersubunit site. Epi-PREG binding to the intersubunit site is consistent with previous findings that Epi-PREG potentiates GABA-elicited currents in cerebellar Purkinje cells and hippocampal pyramidal neurons (Bukanova et al., 2021; M. Wang et al., 2002). We confirmed that Epi-PREG potentiates GABA-elicited currents in α_1_β_3_ receptors, whereas Epi-AlloP and Epi-GX do not. The potentiating effect of Epi-PREG is prevented by the α_1_(Q242L) mutation, further indicating that Epi-PREG binds to the intersubunit NS binding site (Figure 6d).

### 3.8 | The effect of mutations on MQ290 binding

**FIGURE 7:**
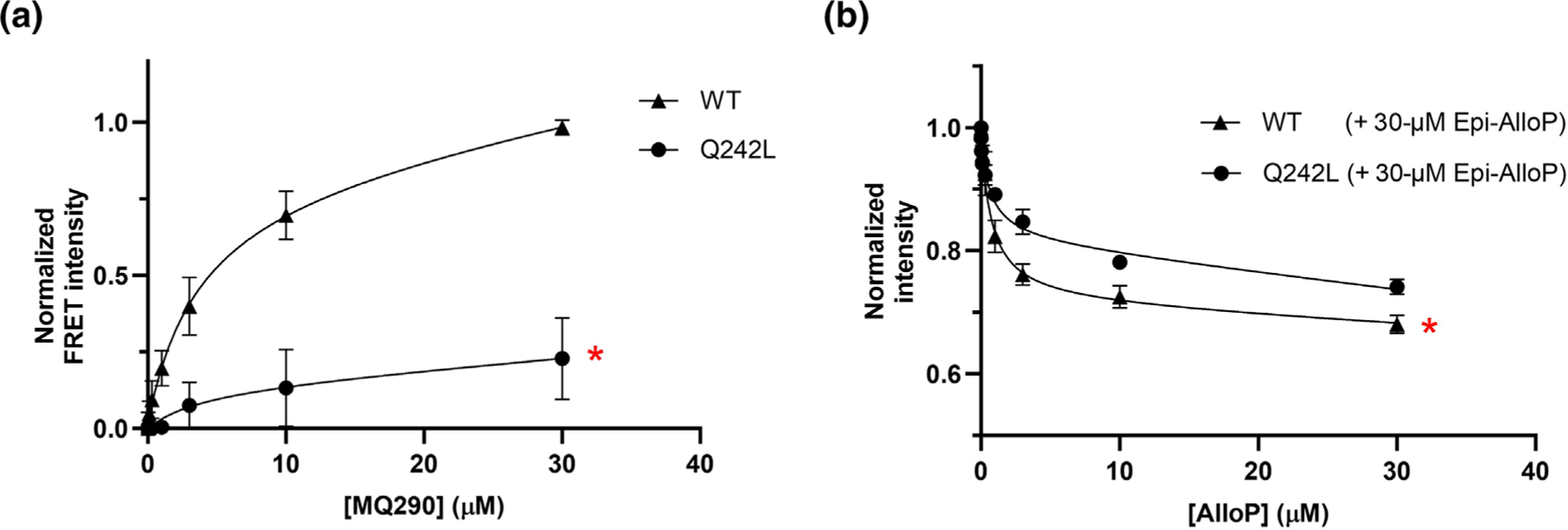
The effect of α_1_^Q242L^ mutation on ELIC-α1GABA_A_R-MQ290 FRET and AlloP binding to the intersubunit site on ELIC-α1GABA_A_R. (a) Concentration–dependent MQ290 FRET signal in ELIC-α1GABA_A_R (WT) and ELIC-α1^Q242L^GABA_A_R (n = 3). The significantly reduced FRET indicates that MQ290 binds to the intersubunit site with a different pose than in WT. (b) AlloP (0.01–30 μM) quenching of tryptophan emission (Ex280) in WT and ELIC-α1^Q242L^GABA_A_R; experiments were conducted in the presence of 30 μM Epi-AlloP to occlude quenching of intrasubunit site tryptophan residues. The data for each sample are normalized to the maximal tryptophan emission (330 nm) in the absence of AlloP (n = 5 ± SE for each data point). The extent of quench is reduced by the Q242L mutation (−17% in Q242L vs. −28% in WT), and there is no change in ${\text{IC}}_{\text{50}}$ values (0.73 ± 0.12 for WT; 0.70 ± 0.18 for Q242L), indicating that Q242L changes the binding pose of AlloP in the intersubunit site with only a modest change in binding affinity. * indicates a difference (*P* < 0.05) in the maximal FRET intensity between WT and Q242L receptors.

The MQ290-NS binding assay provides the ability to distinguish whether mutations that eliminate NS action do so by ablating NS binding or by changing NS binding pose with consequent reduction of efficacy. The Q242L mutation prevents NS potentiation of GABA_A_ currents (Hosie et al., 2006). Photolabelling studies in ELIC-α1GABA_A_R suggest that this mutation does not ablate NS binding, instead causing a NS-analogue photolabelling reagent (KK200) to bind with an inverted orientation (Sugasawa et al., 2019). To confirm that PAM-NS bind to the intersubunit site of ELIC-α_1_^Q242L^GABA_A_Rs and to determine the effect of the mutation on NS affinity, we performed two experiments. First, we analysed concentration-dependent MQ290 FRET in ELIC-α_1_^Q242L^GABA_A_R. The FRET signal was significantly reduced in the Q242L mutants, indicating that MQ290 either does not bind to the intersubunit site or that it binds in a pose in which the B and C rings are not proximal to W246 (Figure 7a). To distinguish between these possibilities and to directly assess authentic NS binding, we measured the ability of AlloP to quench W246 in ELIC-α_1_^Q242L^GABA_A_Rs. Experiments were performed in the presence of 30 μM Epi-AlloP to eliminate contributions from intrasubunit site binding. The maximum quench was significantly reduced in Q242L receptors, but there was no significant change in AlloP binding affinity in comparison to WT (${\text{IC}}_{\text{50}}\;=\;0.73\;\pm \;0.12\;\text{μM}$ in WT vs. 0.70±0.18μM in Q242L) (Figure 7b). These data indicate that the Q242L mutation alters the pose in which AlloP binds in the intersubunit site but has minimal effect on its affinity.

### 3.9 | Mechanism of tryptophan-MQ290 FRET

Energy transfer between tryptophan (donor) and MQ290 (acceptor) could occur either by direct electron exchange (Dexter Resonance Energy Exchange) or via a dipole interaction between the donor and acceptor (Förster Resonance Energy Transfer). The Dexter mechanism requires that the donor and acceptor be in contact (<5 Å proximity) and that the energies of their excited states be the same (Turro et al., 2009). While a Dexter mechanism is plausible because of the known ring stacking interaction of PAM-NS with W246, the calculated energy values of the first excited states of tryptophan and MQ290 (−686 a.u. vs. −929 a.u.) are sufficiently disparate to make a Dexter mechanism unlikely. Assuming a dipole-dependent (Fӧrster) mechanism, we calculated the $R_{0}$ (distance at which the FRET signal decays 50%) for the tryptophan-MQ290 FRET signal (Lakowicz, 2006). The calculated $R_{0}$ value of 5.1 Å is small, enabling measurement of single angstrom changes in the binding pose of MQ290 in the intersubunit site. The distance between the tryptophan donor (W246) and MQ290, was estimated to be <5 Å, consistent with structural studies indicating a ring stacking interaction between 3α-OH NS and W246.

## 4 | DISCUSSION

This study describes the development and application of two direct fluorescence-based binding assays for PAM and NAM-NS binding to specific sites on the α_1_ subunit of GABA_A_ receptors. These assays overcome the challenge of separating free from bound ligand and provide the first reversible assays for measuring binding of NS to allosteric sites in GABA_A_ receptor TMDs. The first assay monitors quenching of tryptophan residues in the NS binding sites by the 17-methylketone group of NS. This is similar to assays previously used to measure low affinity small molecule binding to hydrophobic protein binding sites (Johansson et al., 1995). Quenching requires direct contact (a diffusion radius of ~5 Å) between the NS and a specific tryptophan residue, enabling separate analysis of NS binding to the intersubunit (W246) and α_1_-intrasubunit binding sites (other TMD tryptophans), previously identified by photolabelling (Z.-W. Chen et al., 2019; Sugasawa et al., 2020). Quenching studies showed that AlloP binds with equal affinity (~0.6 μM) to the two binding sites, and Epi-AlloP binds with similar affinity (~0.7 μM) but only to the intrasubunit site. While quenching offers a direct method for studying NS binding, it requires the presence of a ketone group (or other group that acts as an electron acceptor) on the NS and is thus not applicable to screening other ligands for binding to the NS sites.

To develop a more general NS binding assay we synthesized MQ290 as a FRET acceptor for donor tryptophan. Photolabelling studies are consistent with MQ290 binding in the intersubunit site (Figure S5), where it acts as a PAM-NS (Figure 2c,d). The absorption spectrum of MQ290 and the emission spectrum of tryptophan have substantial overlap, enabling resonance energy transfer (Figure 2b). MQ290 exhibits a strong concentration-dependent FRET signal with the tryptophan residues in ELIC-α1GABA_A_R that arises almost completely from the interaction between MQ290 and W246 in the intersubunit site (Figure 3d). Thus, MQ290 is a site-specific reporter of NS binding to the intersubunit site, in contrast to the AlloP quenching assay which reports binding to both the intersubunit and intrasubunit sites. The site specificity of MQ290 could result either from selective binding in the intersubunit site or from a binding pose in the intrasubunit site in which there is insufficient proximity of the triene fluorophore to any tryptophan residue. The small Rₒ value of tryptophan-MQ290 interaction makes either of these mechanisms plausible.

Using MQ290 FRET, we measured the affinity of several 3α-OH NS for the intersubunit NS binding site. Notably, the $K_{i}$ values for AlloP measured using the FRET assay were about 5-fold lower than the $K_{d}$ values obtained with the quench assay, whereas the values for Epi-AlloP were similar with the two assays. One possible explanation for this would be if MQ290 occupies the intrasubunit site producing a negative allosteric effect on NS binding in the intersubunit site. While it would be challenging to test this putative mechanism, the discrepancy between the two assays suggests that there are NS-specific allosteric interactions between NS binding sites on GABA_A_ receptors that remain to be elucidated.

3β-OH NS binding in the intrasubunit site can be indirectly assayed, based on its negative allosteric modulation of MQ290 binding. This allosteric interaction is demonstrated by (1) the observation that the effect of Epi-AlloP on MQ290 $K_{d}$ saturates at high concentration and (2) the absence of Epi-AlloP modulation of FRET in W246L receptors (Figure 4c). The ability to distinguish between competitive and allosteric modulators of ligand binding is a particular strength of the MQ290 binding assay, which should enable mechanistic insights into NS interactions. The allosteric interaction between NS binding in the intersubunit and intrasubunit sites is in seeming conflict with previous electrophysiological studies showing that NS actions at these sites are additive and independent (Germann et al., 2021; L. Wang et al., 2022). The effect of Epi-AlloP binding in the intrasubunit site on MQ290 binding in the intersubunit site is likely due to a subtle allosteric effect that has no detectable effect on NS binding or action but which, due to the exquisite proximity sensitivity of the FRET mechanism, reports on minute differences in the interaction between MQ290 and W246. Consistent with this, the $K_{i}$ of AlloP binding to the intersubunit site is unaffected by Epi-AlloP binding in the intrasubunit site.

NS binding assays based on competitive inhibition of MQ290 FRET demonstrate several aspects of NS pharmacology and highlight potential applications of this method. We screened several 3α-OH NS that act as PAM-NS, demonstrating that while they all compete for binding to the intersubunit site, they exhibit structure-dependent differences in affinity. Interestingly, PREG had significantly lower affinity for the intersubunit site than AlloP, despite their similar concentration-dependent effects in electrophysiological assays. The assay also showed that Epi-PREG, unlike other 3β-OH NS, binds to both the intersubunit and intrasubunit sites (Figure 6b,c). Interestingly, Epi-PREG acts as a PAM at the intersubunit site (Figure 6d), showing that a 3α-OH group is not a strict requirement for NS action as a GABA_A_ receptor PAM. Finally, the MQ290 assay was used to determine the mechanism through which the α_1_Q242L mutation eliminates the PAM effect of NS at the intersubunit site. Prior data suggested that this mutation altered the orientation of KK200, a PAM-NS analogue photolabelling reagent, in the intersubunit site rather than causing a major change in affinity. The MQ290 FRET showed that the Q242L mutation alters the pose of MQ290 and AlloP in the site as manifest by reduced FRET and quench respectively, but that it does not affect AlloP binding affinity. These data indicate that the Q242L mutation eliminates NS efficacy by changing the pose assumed by NS but that hydrogen bonding to Q242 produces only a small contribution to NS binding energy. This information cannot be obtained with functional measurements and is only accessible with a reversible binding assay.

There are several important limitations to the fluorescence-based NS binding assays described in this paper: (1) The requirement for enriched protein. Non-specific interactions of MQ290 with tryptophan residues in native cell membranes largely mask the FRET signal from GABA_A_ receptors, even in membranes from cells overexpressing GABA_A_ receptors. This necessitates performing both the quenching and FRET assays on enriched proteins. (2) Free NS concentrations. The free concentration of NS (either MQ290 or other NS) used to calculate $K_{d}$ is the total NS concentration in the solution. The NS largely partition into the detergent micelles and the free aqueous concentration is not known. This is different than apparent $K_{d}$ values based on electrophysiological measurements, where there is an infinite reservoir of aqueous NS perfusing a cell and the apparent $K_{d}$ is calculated using the aqueous concentration as the free concentration. Thus, $K_{d}$ values measured with the fluorescence assay may not be the same as those estimated from functional studies. The potential discrepancy between apparent $K_{d}$ values and FRET-determined microscopic $K_{d}$ values may be limited by the low efficacy of NS as activators of GABA_A_ currents (Germann et al., 2019). Indeed, the apparent $K_{d}$ value for allopregnanolone determined in α_1_β_3_ GABA_A_ receptors (0.10 ± 0.05 μM) (Tateiwa et al., 2023) is similar to the $K_{i}$ value determined using FRET in ELIC-α1GABA_A_R (0.11 ± 0.02 μM). (3) Use of a chimeric GABA_A_ receptor. We developed these assays using ELIC-α1GABA_A_R because it is readily expressed and purified, stable in detergent and has been shown to have two NS binding sites (Sugasawa et al., 2019). The assay should also work in purified GABA_A_ receptors reconstituted in liposomes or nanodiscs. (4) Based on the lack of effect of cysteamine on MQ290 $K_{d}$ and the desensitized conformation observed for the X-ray crystallographic structure of unliganded ELIC-α1GABA_A_R (Q. Chen et al., 2018), we surmise that the channel is likely to be in either an open or a desensitized state, precluding the ability to observe the effects of orthosteric agonist binding on NS affinity.

We conclude by highlighting two valuable future applications of the methods described here. First, the MQ290 FRET–based assay for NS binding is readily adaptable to a fluorescent plate reader format, enabling screening for high affinity ligands. This will allow identification of compounds that bind with high affinity (disambiguated from their efficacy as agonists) to specific sites. Coupled with functional readouts this should facilitate identification of high affinity agonists, partial agonists and antagonists for the canonical intersubunit NS binding site. Second, development of additional fluorescent NS analogues, for example a 3β-OH fluorescent analogue, should identify site-specific probes for the intrasubunit NS binding sites on GABA_A_ receptors. These probes, in conjunction with MQ290, could be used to screen for site-specific agonists and antagonists for the intrasubunit sites. NS acting at the intrasubunit sites may be more isoform specific than those acting at the intersubunit site (Hosie et al., 2009), holding the prospect of ligands which could be therapeutically useful for treatment of mood disorders and as sedatives and anaesthetics.

## Supplementary Material

Supplementary Material

## Figures and Tables

**TABLE 1:** Binding data for 3α-OH NS and 3β-OH NS ${\text{IC}}_{\text{50}}$ values measured by inhibition of tryptophan–MQ290 FRET.

	${\text{IC}}_{\text{50}}\;\left(\text{μM}\right)$	Hill slope	$K_{i}\;\left(\text{μM}\right)$
a. 3α-OH NS			
AlloP	0.23 ± 0.02	−1.89 ± 0.26	0.11 ± 0.02
PREG	1.92 ± 0.64	−1.02 ± 0.35	0.89 ± 0.30
GX	0.43 ± 0.05	−1.57 ± 0.26	0.20 ± 0.02
YX03	0.91 ± 0.35	−0.98 ± 0.37	0.42 ± 0.
b. 3β-OH NS			
Epi-AlloP	0.36 ± 0.30	−0.68 ± 0.45	0.36 ± 0.30
Epi-PREG	1.31 ± 0.42	−0.77 ± 0.42	1.31 ± 0.42
Epi-GX	1.92 ± 0.40	−0.67 ± 0.30	1.92 ± 0.40

*Note*: (a) 3α-OH NS data. $K_{i}$ values were calculated using the Cheng-Prusoff equation and an MQ290 $K_{d}$ value of 2.60 μM. (b) 3β-OH NS data. ${\text{IC}}_{\text{50}}$ value is equal to $K_{i}$ based on allosteric mechanism of inhibition. All data n = 5 ± S.E.

## Data Availability

The data that support the findings of this study are available from the corresponding author upon reasonable request.

## What is already known

- 3-Hydroxy neurosteroids can act as either positive or negative allosteric modulators of GABA_A_ receptors.
- There are at least three specific binding sites for 3-hydroxy neurosteroids on GABA_A_ receptors.

## What does this study add

- The study provides methods for directly measuring site-specific binding affinity of neurosteroids on GABA_A_ receptors.

## What is the clinical significance

- These methods will enable identification of high-affinity, site-specific ligands for neurosteroid binding sites.
- Site-specific GABA_A_ modulators will be useful in treating mood disorders and as sedatives and anaesthetics.

## Abbreviations:

AlloP: allopregnanolone
ELIC: *Erwinia chrsysanthemi* ligand-gated ion channel
FRET: Fӧrster resonance energy transfer
NS: neurosteroid
PET: photo-induced electron transfer
PREG: pregnanolone
TMD: transmembrane domain
