# Supplementary material for: Direct measurements of neurosteroid binding to specific sites on GABAA receptors
Source: Br J Pharmacol. Author manuscript; Available in PMC 2024 Nov 24. (PMC11585924; doi:10.1111/bph.16490)
Supplement: Supplementary Material [file NIHMS2031446-supplement-Supplementary_Material.pdf]

Supplemental Figures

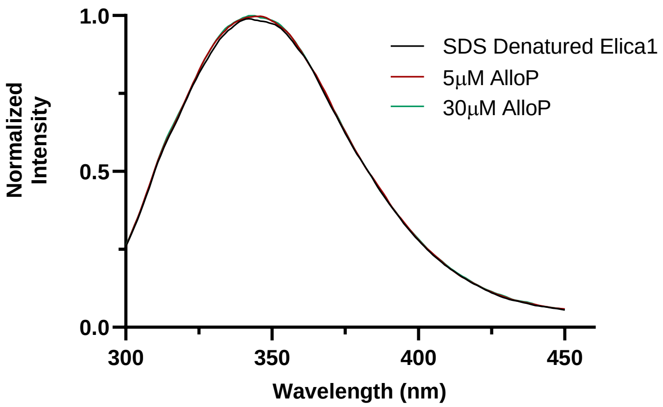

Supplemental Figure 1. ***AlloP quenching is dependent on the conformational integrity of ELIC-α1GABA$_A$R.*** Tryptophan emission spectra (Ex280 nm) of SDS-denatured ELIC-α1GABA$_A$R in the presence and absence of AlloP (5 and 30 μM). The absence of the AlloP quenching observed in intact ELIC-α1GABA$_A$R (Figure 1c) indicates that an appropriately folded protein is required for AlloP binding and quenching.

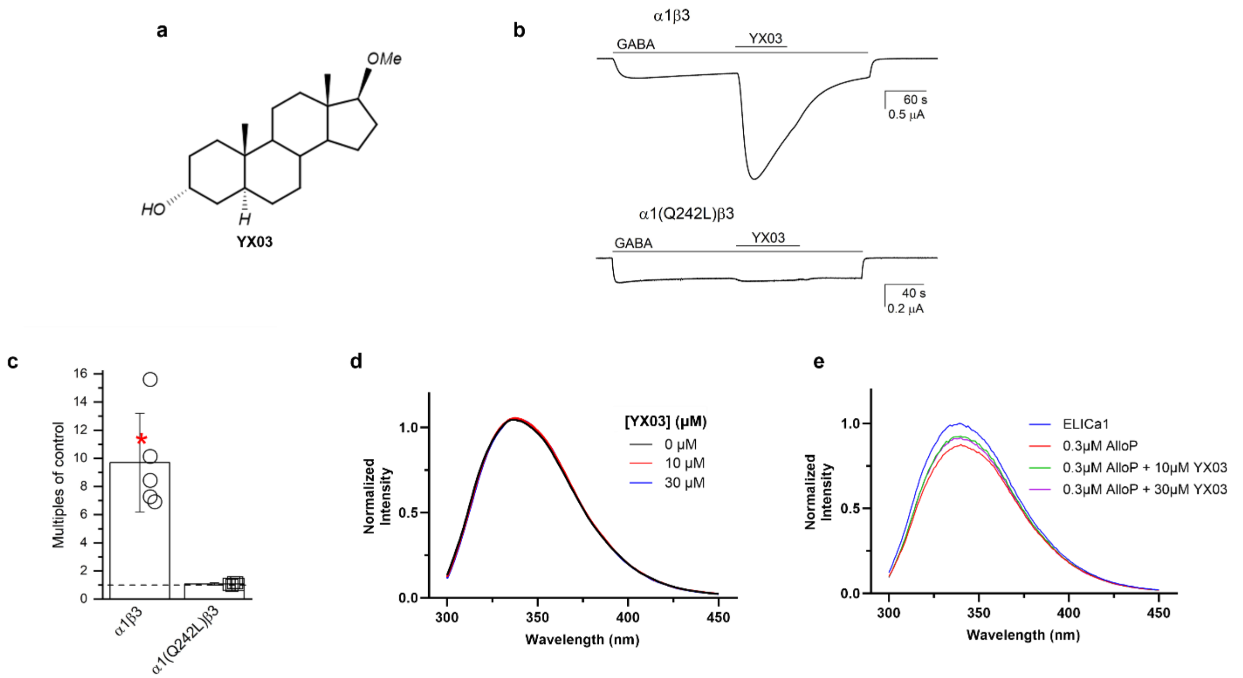

Supplemental Figure 2. ***Quenching of Tryptophan emission of ELIC-α1GABA$_A$R is absent for YX03, a 3α-OH NS lacking the 17-methylketone on the steroid D-ring.*** (**a**) Structure of YX03 with a 17-methoxy group replacing the 17-methylketone in AlloP. (**b, c**) YX03 is a positive allosteric modulator of $\alpha_1\beta_3$ GABA$_A$ receptors, acting at the intersubunit binding site. (**b**) Representative tracing showing YX03 (1 μM) potentiation of currents elicited by low GABA in $\alpha_1\beta_3$ GABA$_A$ receptors expressed in *Xenopus* oocytes. (**c)** Enhancement of currents elicited with low GABA (n = 5) by YX03 (1 μM) in $\alpha_1\beta_3$ (○- open circles) and $\alpha_1^{Q241L}\beta_3$ (□ – open squares) ($\alpha_1\beta_3$ vs. $\alpha_1^{Q241L}\beta_3$, $p < .001$). The y-axis shows the ratio of the response to GABA + YX03 vs. GABA alone, with a value of 1 indicating no enhancement. * Indicates that in $\alpha_1\beta_3$, but not $\alpha_1^{Q242L}\beta_3$ receptors, YX03 enhances ($p < 0.05$) the currents elicited by GABA. (**d**) Tryptophan emission spectra (Ex280 nm) of ELIC-α1GABA$_A$R in the presence and absence of YX03 (10 and 30 μM). The absence of quenching by YX03 (as compared to the quenching observed with AlloP (Figure 1c)) indicates that the 17-methylketone is required for bound AlloP quenching.

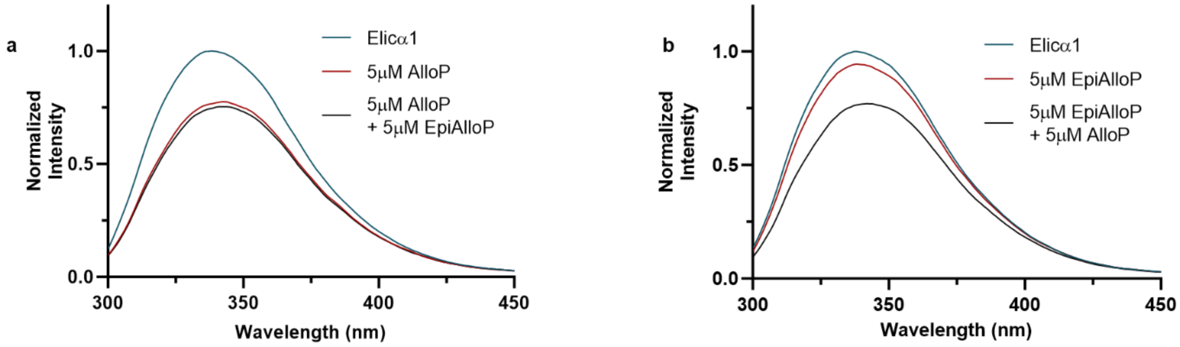

Supplemental Figure 3. ***Epi-AlloP binds to a subset of AlloP binding sites on ELIC-α1GABA$_A$R***. Fluorescence emission spectra (Ex280 nm) of ELIC-α1GABA$_A$R (0.3 μM) in the presence of indicated concentrations of AlloP and Epi-AlloP. (**a**) A saturating concentration of Epi-AlloP (5μM) does not add to the quenching produced by a saturating concentration of AlloP (5μM). (**b**) A saturating concentration of AlloP (5μM) increases the quenching produced by a saturating concentration of Epi-AlloP (5μM).

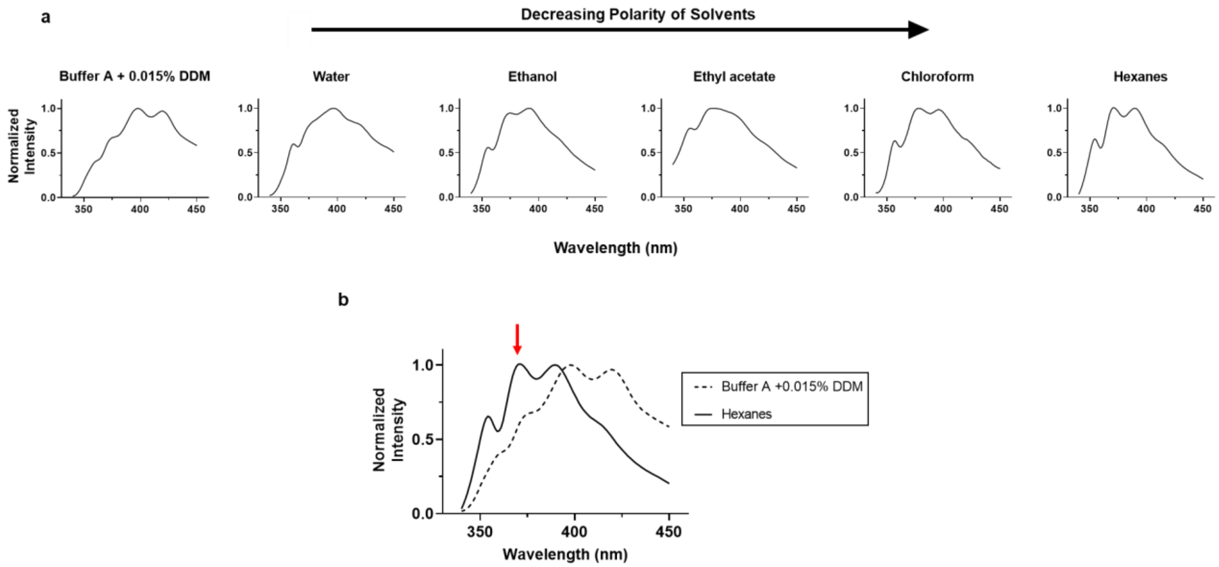

Supplemental Figure 4. ***MQ290 fluorescence emission maximum varies with solvent polarity.*** (**a**) Fluorescence emission spectra (Ex330 nm) of MQ290 (10 µM) in solvents of decreasing polarity. There is a blue-shift in emission maxima from 400 nm in aqueous solvent to 370 nm in hexanes. All spectra are normalized to a maximal peak intensity of 1. (**b**) Superimposed spectra of MQ290 in hexanes and detergent-containing buffer demonstrating the maximum difference between the spectra at 370 nm (red arrow). FRET signals were quantified at 370 nm to monitor MQ290 in the protein-bound (non-polar) environment.

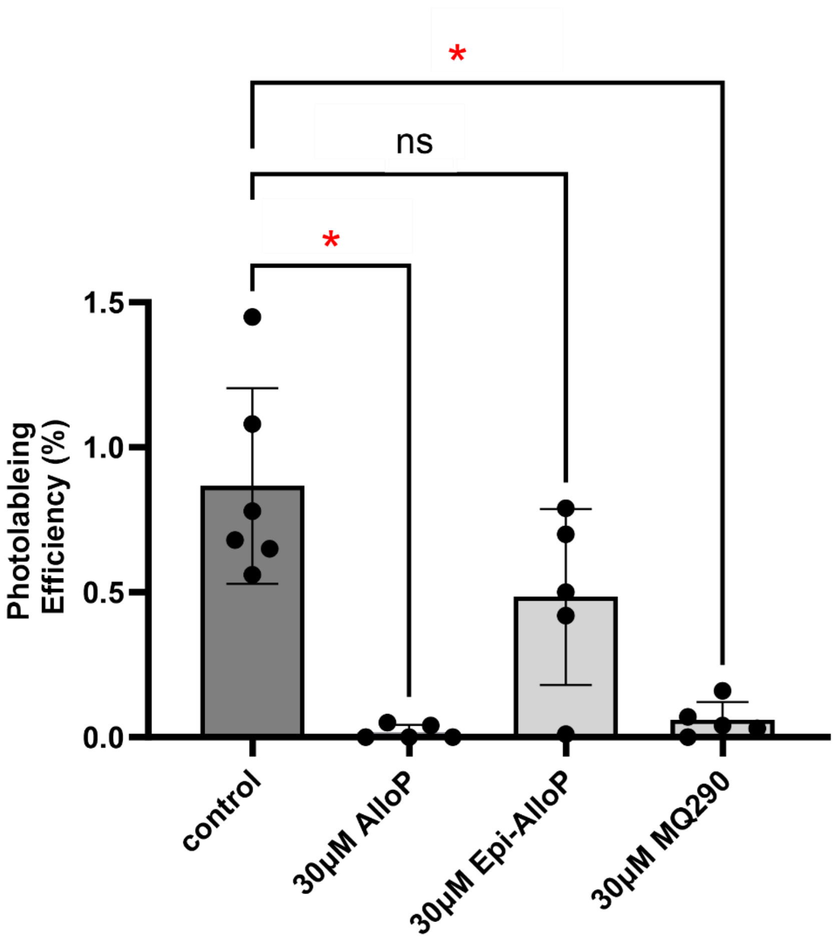

Supplemental Figure 5. ***Inhibition of NS analogue (KK200) photolabeling of the intersubunit NS binding site on ELIC-α1GABA$_A$R.*** Photolabeling efficiency of TM3 (Y309) by 3 μM KK200 in ELIC-α1GABA$_A$R in the absence (control) or presence of 30 μM AlloP, Epi-AlloP or MQ290.There was a statistically significant difference between groups as determined by one-way ANOVA ($F = 15.65$, $p < 0.0001$). Tukey's post-hoc multiple comparison test of the means showed that the effects of ALLO and MQ290 were significantly different than control ($p < 0.0002$ vs. control for each comparison) whereas the effect of Epi-AlloP was not significantly different than control. Data are shown as mean ± SD with n = 5 for each point. Statistical differences: * = $p < 0.05$; ns = not significant.

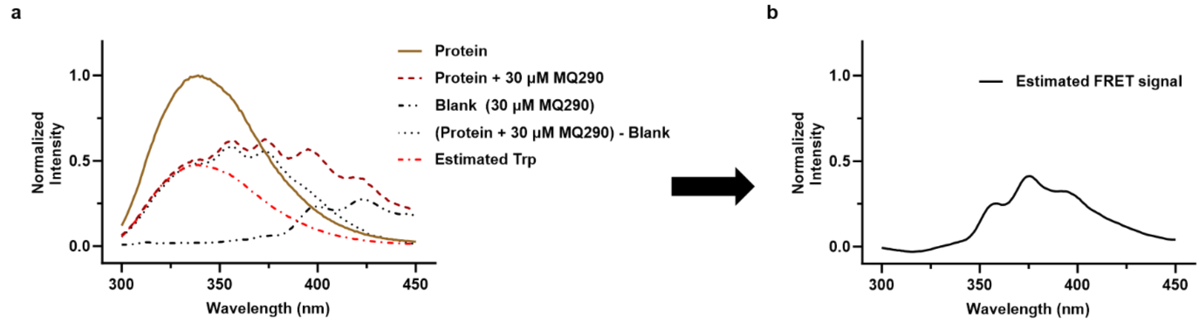

Supplemental Figure 6. ***Extraction of MQ290 FRET signal***. To measure the FRET spectrum resulting from the energy transfer from donor tryptophan to bound MQ290, the spectrum of MQ290 in sample buffer was subtracted from the spectrum of protein + MQ290; the estimated spectrum of residual tryptophan emission was then subtracted to provide the FRET spectrum. (**a**) Fluorescence emission spectra of: ELIC-α1GABA$_A$R in the absence of MQ290 (Protein); ELIC-α1GABA$_A$R in the presence of MQ290 (Protein + MQ290); MQ290 alone (Blank); Subtraction of spectra: ((protein+ MQ290) – Blank) and; estimated contribution of tryptophan emission to ELIC-α1GABA$_A$R + MQ290 spectrum (Estimated Trp). (**b**) MQ290- ELIC-α1GABA$_A$R FRET spectrum calculated by subtraction of the residual tryptophan spectrum (Estimated Trp) and the MQ290 buffer spectrum from the spectrum of ELIC-α1GABA$_A$R in the presence of MQ290 (Protein + MQ290) as shown in Panel a.

Supplemental Methods

Synthetic Procedure for MQ290

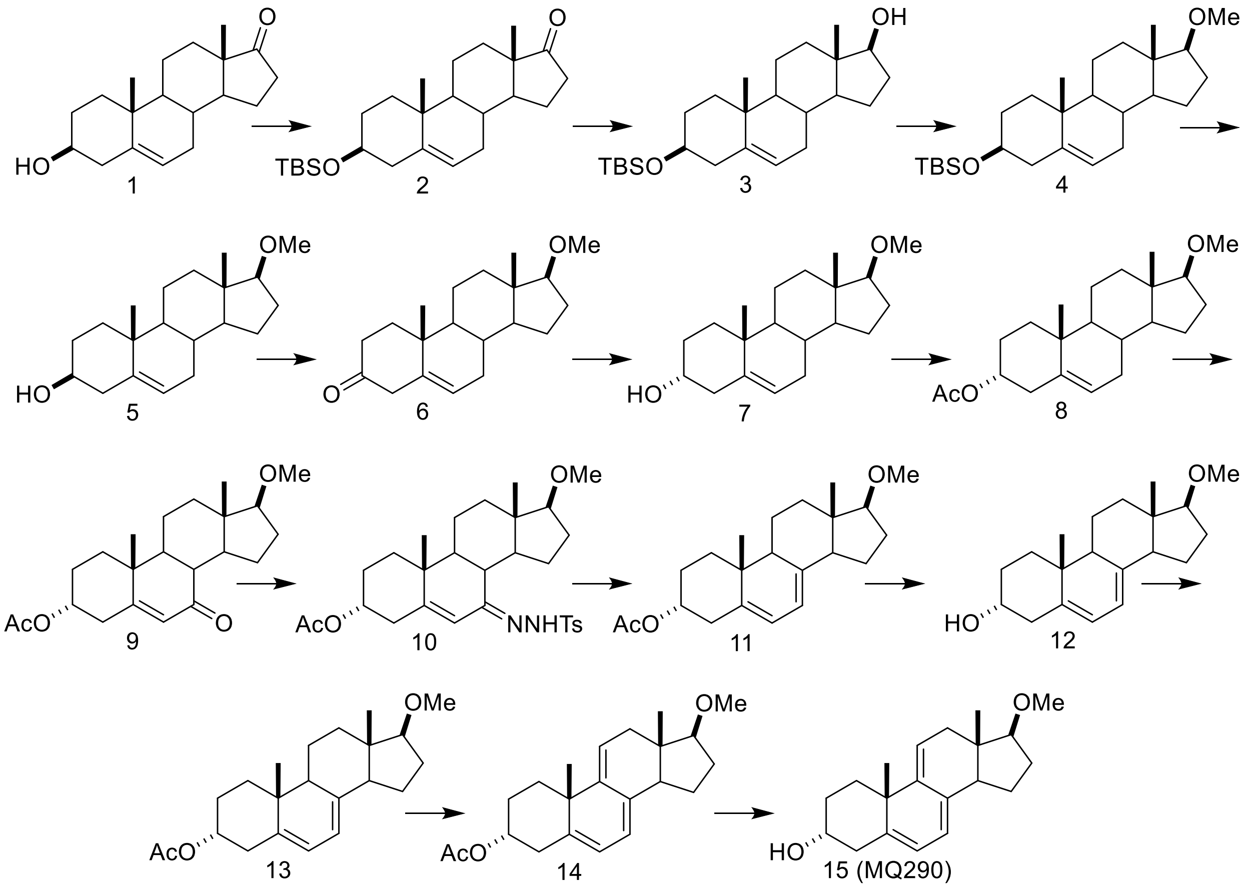

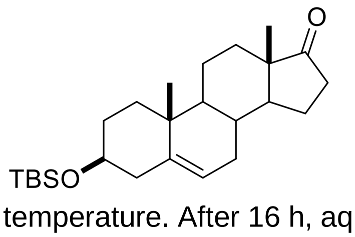

(3β)-3-[[(1,1-Dimethylethyl)dimethylsilyl]oxy]androst-5-en-17-one (2). To a solution of (3β)-3-hydroxyandrost-5-en-17-one (**1**, 2.4 g, 8.3 mmol) in DMF (50 mL) was added *tert*-butyldimethylsilyl chloride (1.9 g, 12.5 mmol) and imidazole (1.36 g, 20 mmol) at room temperature. After 16 h, aqueous $NaHCO_3$ was added and the product was extracted into EtOAc (200 mL). The organic layer was washed with brine (100 mL x3). The solvent was removed and the residue was purified by flash chromatography (silica gel, eluted with 10% EtOAc in hexanes)

to give steroid **2** (3.35 g, 100%): ^{1}H NMR (400 MHz, $CDCl_3$) δ 5.36-5.34 (m, 1H), 3.53-3.45 (m, 1H), 2.50-0.96 (m, 19H), 1.03 (s, 3H), 0.89 (s, 12H), 0.06 (s, 6H); ^{13}C NMR (100 MHz, $CDCl_3$) δ 221.2, 141.7, 120.4, 72.4, 51.7, 50.2, 47.5, 42.7, 37.3, 36.7, 35.0, 32.0, 31.5, 31.4, 30.8, 25.9 (3 x C), 21.8, 20.3, 19.4, 18.2, 13.5, -4.6 (2 x C).

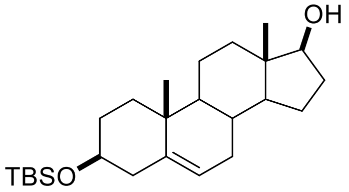

(3β,17β)-3-[[(1,1-Dimethylethyl)dimethylsilyl]oxy]androst-5-en-17-ol (3). To a solution of steroid **2** (3.35 g, 8.33 mmol) in EtOH (100 mL) was added $NaBH_4$ (800 mg, 20 mmol) at room temperature. After 2 h, aqueous $NaHCO_3$ (60 mL) was added and stirring continued for 30 min. The product was extracted into EtOAc (250 mL). The solvent was removed and the residue was purified by flash chromatography (silica gel, eluted with 10-20% EtOAc in hexanes) to give steroid **3** (3.08 g, 92%): ^{1}H NMR (400 MHz, $CDCl_3$) δ 5.33-5.32 (m, 1H), 3.68 (t, *J* = 8.6 Hz, 1H), 3.53-3.45 (m, 1H), 2.32-0.93 (m, 20H), 1.03 (s, 3H), 0.90 (s, 9H), 0.77 (s, 3H), 0.07 (s, 6H); ^{13}C NMR (100 MHz, $CDCl_3$) δ 141.6, 120.8, 81.8, 72.5, 51.3, 50.3, 42.8, 42.7, 37.4, 36.6, 36.5, 32.0, 31.9, 31.5, 30.5, 25.9 (3 x C), 23.4, 20.6, 19.4, 18.2, 10.9, -4.6 (2 x C).

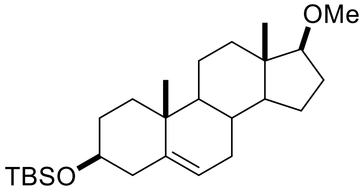

(3β)-3-[[(1,1-Dimethylethyl)dimethylsilyl]oxy]-17-methoxyandrost-5-ene (4). To a solution of steroid **3** (3.08 g, 7.62 mmol) in THF (60 mL) was added NaH (60% in mineral oil, 1.2 g, 30 mmol) at room temperature. The mixture was refluxed for 1 h, iodomethane (40 mmoL) was added and the reaction was refluxed for 16 h. After cooling to room temperature, aqueous $NaHCO_3$ was slowly added and the product was extracted into EtOAc (150 mL x 2). The solvent was removed and the residue was purified by flash chromatography (silica gel, eluted with 10% EtOAc in hexanes) to give steroid **4** (2.96 g, 93%): ^{1}H NMR (400 MHz, $CDCl_3$) δ 5.33-5.32 (m, 1H), 3.52-3.47 (m, 1H), 3.37 (s, 3H), 3.27 (t, *J* = 8.1 Hz, 1H), 2.32-0.85 (m, 19H), 1.02 (s, 3H), 0.91 (s, 9H), 0.79 (s, 3H), 0.07 (s, 6H); ^{13}C NMR (100 MHz, $CDCl_3$) δ 141.5, 120.8, 90.7, 72.5, 57.8, 51.6, 50.3, 42.8, 42.6, 37.9, 37.4, 36.6, 32.0, 31.7, 31.5, 27.6, 25.9 (3 x C), 23.3, 20.7, 19.4, 18.2, 11.4, -4.6 (2 x C).

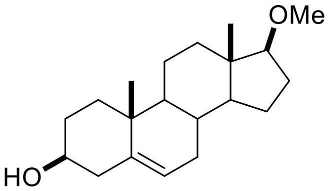

(3β,17β)-17-Methoxyandrost-5-en-3-ol (5). To a solution of steroid **4** (349 mg, 0.74 mmol) in THF (20 mL) was added TBAF (1.0 M in THF, 2 mL, 2 mmol) at room temperature. After 16 h, THF was removed, and the residue was purified by flash chromatography (silica gel, eluted with 20% EtOAc in hexanes) to give steroid **5** (205 mg, 83%): ^{1}H NMR (400 MHz, $CDCl_3$) δ 5.31-5.30 (m, 1H), 3.51-3.43 (m,

1H), 3.32 (s, 3H), 3.22-3.18 (m, 1H), 2.47 (s, 1H), 2.28-0.87 (m, 20H), 0.98 (s, 3H), 0.74 (s, 3H); ^{13}C NMR (100 MHz, $CDCl_3$) δ 140.8, 121.1, 90.6, 71.4, 57.7, 51.4, 50.0, 42.5, 42.1, 37.7, 37.1, 36.4, 31.5, 31.4, 31.3, 27.5, 23.2, 20.6, 19.3, 11.3.

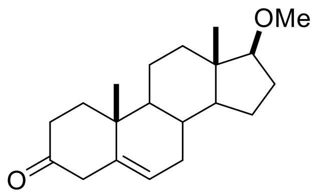

(17β)- 17-Methoxyandrost-5-en-3-one (6). To a solution of steroid **5** (1.7 g, 5.6 mmol) in dichloromethane (50 mL) was added Dess-Martin Periodinane (3.56 g, 8.4 mmol) at room temperature. After 2 h, water was added and the product was extracted into dichloromethane (150 ml x 2). The organic layer was washed with brine (50 mL). The solvent was removed, and the residue was purified by flash chromatography (silica gel eluted with 10-25% EtOAc in hexanes) to give steroid **6** (1.23 g, 72%): ^{1}H NMR (400 MHz, $CDCl_3$) δ 5.21-5.20 (m, 1H), 3.22 (s, 3H), 3.18-3.09 (m, 2H), 2.70-0.71 (m, 18H), 1.07 (s, 3H), 0.67 (s, 3H); ^{13}C NMR (100 MHz, $CDCl_3$) δ 209.5, 138.4, 122.2, 90.2, 57.5, 51.1, 49.0, 48.0, 42.4, 37.4, 37.3, 36.6, 36.6, 31.4, 31.1, 27.3, 23.1, 20.7, 18.9, 11.2.

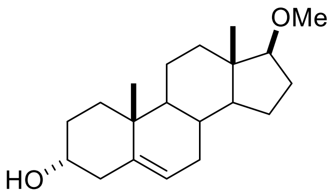

(3α,17β)-17-Methoxyandrost-5-en-3-ol (7). To a solution of steroid **6** (1.23 g, 4.1 mmol) in THF (80 mL) was added K-selectride (1.0 M in THF, 6 mL, 6 mmol) at -78 °C. After 2 h, 3 N NaOH (10 mL) and H_2O_2 (3 mL) were added at -78 °C. After addition, the reaction was warmed to room temperature for 1 h. The product was extracted into EtOAc (100 mL x 3). The combined organic layers were washed with brine (50 mL x 3) and water (100 mL). The solvent was removed and the residue was purified by flash chromatography (silica gel, eluted with 20% EtOAc in hexanes) to give steroid **7** (877 mg 71%): ^{1}H NMR (400 MHz, $CDCl_3$) δ 5.38-5.37 (m, 1H), 3.99 (s, 1H), 3.33 (s, 3H), 3.22 (t, J = 8.2 Hz, 1H), 2.56-2.52 (m, 1H), 2.07-0.78 (m, 19H), 0.99 (s, 3H), 0.75 (s, 3H); ^{13}C NMR (100 MHz, $CDCl_3$) δ 138.6, 123.3, 99.6, 66.8, 57.8, 51.4, 50.2, 42.5, 39.7, 37.7, 37.2, 33.0, 31.5, 31.4, 28.7, 27.5, 23.2, 20.3, 18.6, 11.3.

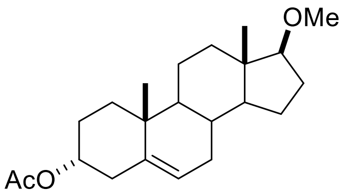

(3α,17β)-17-Methoxyandrost-5-en-3-ol, acetate (8). To a solution of steroid **7** (785 mg, 2.58 mmol) in dichloromethane (20 ml) was added acetic anhydride (0.57 mL, 6 mmol), Et_3N (1.26 mL, 9 mmol), and DMAP (20 mg) at room temperature. After 16 h, the solvent was removed, and the residue was purified by flash chromatography (silica gel, eluted with 10-20% EtOAc in hexanes) to give steroid **8** (782 mg 87%): ^{1}H NMR (400 MHz, $CDCl_3$) δ 5.28-5.27 (m, 1H), 4.99 (s, 1H), 3.36 (s, 3H), 3.26 (t, J = 8.6 Hz, 1H), 2.50-2.47 (m, 1H), 2.23-0.99 (m, 18H),

2.02 (s, 3H), 1.02 (s, 3H), 0.78 (s, 3H); ^{13}C NMR (100 MHz, $CDCl_3$) δ 170.8, 138.5, 121.8, 90.7, 70.5, 57.8, 51.6, 49.9, 42.6, 37.9, 37.0, 36.2, 33.4, 31.6, 31.4, 27.6, 26.1, 23.3, 21.4, 20.4, 18.9, 11.4.

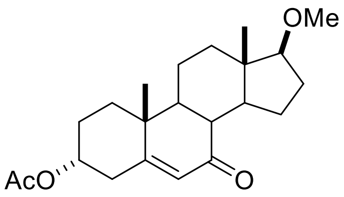

(3α,17β)-3-(Acetyloxy)-17-methoxyandrost-5-en-7-one (9). Chromium trioxide (4.46 g, 45 mmol) was suspended in dry dichloromethane (30 mL) at -20 °C and dimethyl pyrazole (4.32 g, 45 mmol) was added in one portion. After stirring at -20 °C for 30 min, steroid **8** (782 mg, 2.25 mmol) in dichloromethane (10 mL) was added. After stirring for 4 h and maintaining the temperature between -10 °C and -20 °C, the reaction mixture was filtered through Celite and washed with dichloromethane (150 mL). The solvent was removed and the residue was purified by flash chromatography (silica gel, eluted with 10-20% EtOAc in hexanes) to give steroid **9** (419 mg 52%): ^{1}H NMR (400 MHz, $CDCl_3$) δ 5.66 (s, 1H), 5.12 (s, 1H), 3.34 (s, 3H), 3.25 (t, *J* =7.8 Hz, 1H), 2.63-2.59 (m ,1H), 2.45-1.12 (m, 16H), 2.00 (s, 3H), 1.21 (s, 3H), 0.77 (s, 3H); ^{13}C NMR (100 MHz, $CDCl_3$) δ 201.6, 170.4, 164.1, 126.8, 89.7, 69.6, 57.7, 49.8, 45.1, 45.0, 43.2, 38.7, 36.6, 36.3, 32.9, 27.6, 25.7, 25.6, 21.2, 20.5, 16.9, 11.6.

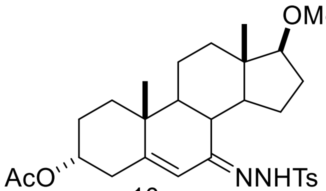

10

4-Methylbenzenesulfonic acid [(3α,17β)-3-(acetyloxy)-17-methoxyandrost-5-en-7-ylidene]hydrazide (10). Steroid **9** (419 mg, 1.16 mmol) and *p*-toluenesulfonyl hydrazide (4.3 g, 23 mmol) were dissolved in methanol (50 mL). The reaction was refluxed for 5 h. After cooling to room temperature the reaction was stirred for 16 h. The solvent was removed, and the residue was purified by flash chromatography (silica gel, eluted with hexanes/DCM/EtOAc = 8/1/1 to 8/1.5/1.5) to give crude steroid **10** (750 mg crude weight also containing some unreacted steroid **9**): ^{1}H NMR (400 MHz, $CDCl_3$) δ 7.80 (d, *J* = 8.8 Hz, 2H), 7.27 (d, *J* = 8.8 Hz, 2H), 5.93 (s, 1H), 5.00 (s, 1H), 3.29 (s, 3H), 3.15 (t, *J* = 8.1 Hz, 1H), 2.52-1.04 (m, 18H), 1.98 (s, 3H), 1.90 (s, 3H), 1.02 (s, 3H), 0.67 (s, 3H); ^{13}C NMR (100 MHz, $CDCl_3$) δ 170.6, 156.3, 155.4, 143.7, 143.6, 135.4, 129.2 (2 x C), 128.0 (2 x C), 113.5, 89.7, 69.7, 60.3, 57.6, 49.1, 45.2, 42.8, 38.9, 38.8, 36.5, 36.3, 27.3, 26.1, 25.1, 21.4, 20.0, 14.0, 11.7.

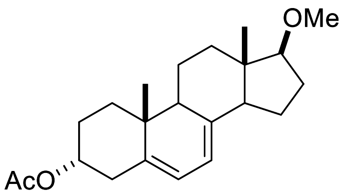

(3α,17β)-17-Methoxyandrosta-5,7-dien-3-ol, acetate (11). Lithium hydride (184 mg, 23 mmol) was added to a solution of crude steroid **10** (750 mg) in anhydrous toluene (60 mL) and the reaction mixture was refluxed for 1 h under N_2. The reaction was cooled and water (30 mL) was slowly added. The organic layer was separated, and the aqueous layer was extracted with EtOAc (60 mL x 3). The combined organic layers were washed with brine (50 mL). The solvent was removed and the residue was purified by flash chromatography (silica gel, eluted with 0-10% EtOAc in hexanes) to give steroid **11** (185 mg containing a small amount of a Δ^5-7-benzyl substituted byproduct): ^{1}H NMR (400 MHz, $CDCl_3$) δ 5.49-5.48 (m, 1H), 5.35 (s, 1H), 5.00 (s, 1H), 3.31 (s, 3H), 3.29 (t, *J* = 7.8 Hz, 1H), 2.49-0.72 (m, 16H), 1.97 (s, 3H), 0.89 (s, 3H), 0.65 (s, 3H); ^{13}C NMR (100 MHz, $CDCl_3$) δ 170.8, 139.9, 138.0, 119.0, 116.6, 90.4, 69.3, 57.8, 49.2, 45.8, 43.0, 37.3 (2 x C), 34.8, 33.8, 27.5, 26.0, 22.0, 21.4, 20.3, 15.9, 11.5.

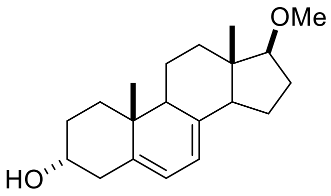

(3α,17β)-17-Methoxyandrosta-5,7-dien-3-ol (12). To a solution of steroid **11** (185 mg, 0.54 mmol) in methanol (20 mL) was added K_2CO_3 (500 mg) at room temperature. The mixture was refluxed for 2 h, methanol was removed and the residue was purified by flash chromatography (silica gel, eluted with 10-20% EtOAc in hexanes) to give steroid **12** (134 mg, 83%). The contaminating Δ^5-7-benzyl substituted byproduct contained in steroid **11** was removed during purification of steroid **12** which had: ^{1}H NMR (400 MHz, $CDCl_3$) δ 5.59 (s, 1H), 5.38 (s, 1H), 4.07 (s, 1H), 3.36 (s, 3H), 3.34 (t, *J* = 7.8 Hz, 1H), 2.58-1.24 (m, 17H), 0.93 (s, 3H), 0.69 (s, 3H); ^{13}C NMR (100 MHz, $CDCl_3$) δ 140.2, 137.8, 121.2, 116.5, 90.4, 66.0, 57.9, 49.2, 46.1, 43.0, 38.3, 37.4, 37.3, 33.6, 28.8, 27.5, 22.0, 20.4, 15.9, 11.4.

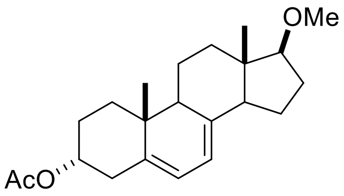

(3α,17β)-17-Methoxyandrosta-5,7-dien-3-ol , acetate (13). To a solution of steroid **12** (134 mg, 0.44 mmol) in dichloromethane (20 mL) was added acetic anhydride (0.28 mL, 3 mmol), Et_3N (0.7 mL, 5 mmol), and DMAP (15 mg) at room temperature. After 16 h, the solvent was removed under reduced pressure and the residue was purified by flash chromatography (silica gel, eluted with 10% EtOAc in hexanes) to give steroid **13** (126 mg, 82%): ^{1}H NMR (400 MHz, $CDCl_3$) δ 5.55-5.54 (m, 1H), 5.41 (s, 1H), 5.06 (s, 1H), 3.38 (s, 3H), 3.36 (t, *J* = 7.4 Hz, 1H), 2.56-1.26 (m, 16H), 2.03 (s, 3H), 0.96 (s, 3H), 0.72 (s, 3H); ^{13}C NMR (100 MHz, $CDCl_3$) δ 170.8, 140.0, 138.1, 119.8, 116.6, 90.4, 69.4, 57.9, 49.2, 45.8, 43.0, 37.3, 37.3, 34.8, 33.8, 27.5, 26.0, 22.0, 21.4, 20.3, 15.9, 11.5.

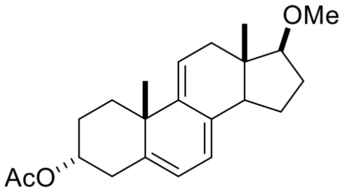

(3α,17β)-17-Methoxyandrosta-5,7,9(11)-trien-3-ol , acetate (14). Mercuric acetate (1.15 g, 3.6 mmol) was stirred in acetic acid (18 mL) for 15 min. A solution of steroid **13** (126 mg, 0.37 mmol) in dichloromethane (12 mL) was added and the reaction mixture was stirred at room temperature for 18 h. The mixture was added to water (30 mL) and the product was extracted into diethyl ether (200 mL). The organic layer was washed with brine (50 mL x 2) and aqueous $NaHCO_3$ (60 mL). The solvent was removed and the residue was purified by flash chromatography (silica gel, eluted with hexanes/ethyl acetate/Et_3N = 100/20/1) to give steroid **14** (56 mg, 44%): ^{1}H NMR (400 MHz, $CDCl_3$) δ 5.63-5.62 (m, 1H), 5.55-5.54 (m, 1H), 5.42-5.41 (m, 1H), 5.07 (s, 1H), 3.46 (t, *J* = 7.8, 1H), 3.38 (s, 3H), 2.75-2.71 (m, 1H), 2.38-1.55 (m, 12H), 2.02 (s, 3H), 1.27 (s, 3H), 0.68 (s, 3H); ^{13}C NMR (100 MHz, $CDCl_3$) δ 170.8, 144.8, 140.5, 134.2, 121.2, 118.8, 115.9, 90.6, 71.5, 57.8, 46.0, 42.3, 41.0, 40.0, 35.7, 35.0, 30.2, 28.1, 26.6, 21.9, 21.4, 11.0.

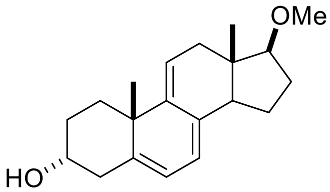

(3α,17β)-17-Methoxyandrosta-5,7,9(11)-trien-3-ol (15, MQ290). To a solution of steroid **14** (56 mg, 0.16 mmol) in methanol (10 mL) was added K_2CO_3 (200 mg) at room temperature. The mixture was refluxed for 2 h, methanol was removed under reduced pressure, and the residue was purified by flash chromatography (silica gel, eluted with hexanes/ethyl acetate/Et_3N = 100/20/1) to give steroid **15**, **MQ290** (36 mg, 73%): mp. 170-172 °C; $[\alpha]_D^{20}$ +93.3 (*c* = 0.06, $CHCl_3$); ^{1}H NMR (400 MHz, $CDCl_3$) δ 5.74-5.72 (m, 1H), 5.55-5.54 (m, 1H), 5.41-5.40 (m, 1H), 4.09 (s, 1H), 3.46 (t, *J* = 8.2, 1H), 3.38 (s, 3H), 2.83-2.80 (m,1H), 2.39-1.33 (m, 13H), 1.28 (s, 3H), 0.67 (s, 3H); ^{13}C NMR (100 MHz, $CDCl_3$) δ 144.7, 140.5, 134.7, 121.5, 120.1, 115.5, 90.6, 68.6, 57.8, 46.0, 42.4, 41.0, 40.3, 39.4, 35.6, 29.7, 29.6, 28.1, 21.8, 11.0.

Synthetic Procedures for YX03

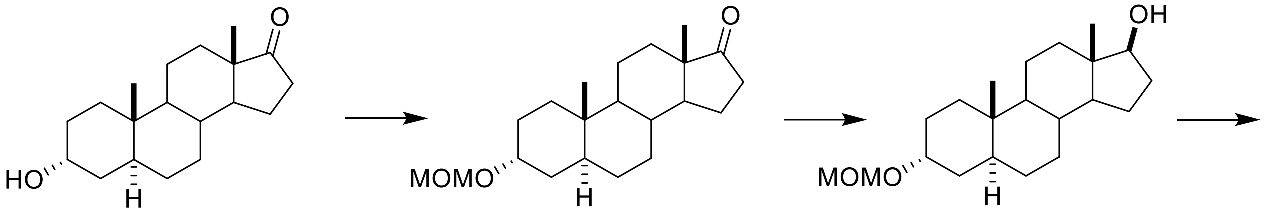

16 17

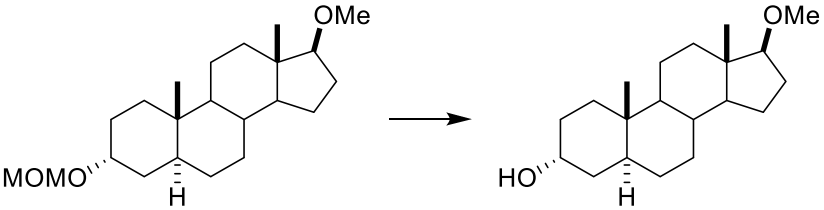

18 19 (YX03)

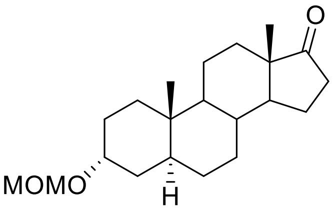

(3α)-3-[[(1,1-Dimethylethyl)dimethylsilyl]oxy]androstan-17-one (16). To a solution of androsterone (510 mg, 1.76 mmol) in dichloromethane (15 mL) was added chloromethyl methyl ether (5.28 mmol) and (*i*-Pr)$_2$NEt at room temperature. After 16 h, the solvent was removed and the residue was purified by flash chromatography (silica gel, eluted with 20% EtOAc in hexanes) to give steroid **16** (580 mg , 99%): ^{1}H NMR (400 MHz, $CDCl_3$) δ 4.67-4.63 (m, 2H), 3.83 (s, 1H), 3.36 (s, 3H), 2.46-2.39 (m, 1H), 2.08-2.01 (m, 1H), 1.94-0.79 (m, 20H), 0.85 (s, 3H), 0.81 (s, 3H); ^{13}C NMR (100 MHz, $CDCl_3$) δ 221.4, 94.5, 71.5, 55.1, 54.4, 51.5, 47.8, 39.7, 36.0, 35.8, 35.0, 33.6, 32.8, 31.5, 30.8, 28.3, 26.2, 21.7, 20.0, 13.8, 11.4.

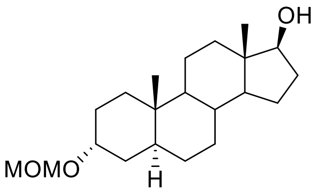

(3α,17β)-3-[[(1,1-Dimethylethyl)dimethylsilyl]oxy]androstan-17-ol (17). To a solution of steroid **16** (580 mg, 1.74 mmol) in ethanol (30 mL) was added sodium borohydride (300 mg, 7.5 mmol) at room temperature. After 2 h, aqueous NH_4Cl was added and the ethanol was removed. Water (10 mL) was added and the product was extracted into dichloromethane (30 mL x 3). The solvent was removed and the residue was purified by flash chromatography (silica gel, eluted with 20% EtOAc in hexanes) to give steroid **17** (580 mg, 99%): ^{1}H NMR (400

MHz, $CDCl_3$) δ 4.65 (m, 2H), 3.82 (s, 1H), 3.62 (t, J = 8.2 Hz, 1H), 3.36 (s, 3H), 2.09-1.99 (m, 1H), 1.80-0.85 (m, 22H), 0.79 (s, 3H), 0.73 (s, 3H); ^{13}C NMR (100 MHz, $CDCl_3$) δ 94.5, 81.9, 71.6, 55.1, 54.4, 51.1, 43.0, 39.8, 36.7, 35.9, 35.5, 33.6, 32.8, 31.5, 30.5, 28.4, 26.3, 23.3, 20.3, 11.4, 11.1.

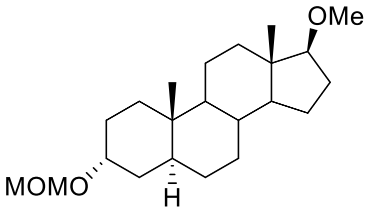

(3α,17β)-3-[[(1,1-Dimethylethyl)dimethylsilyl]oxy]-17-methoxyandrostane (18). A suspension of the sodium hydride (60% in mineral oil, 0.88 g, 22 mmol) and steroid **17** (580 mg, 1.74 mmol) in THF (30 mL) was refluxed under N_2 for 1 h. Iodomethane (3.1 mL, 50 mmol) was added and the mixture was stirred for an additional 2 h. After cooling to room temperature, water was slowly added and the product was extracted into EtOAc (100 ml x 2, 50 mL). The solvent was removed and the residue was purified by flash chromatography (silica gel, eluted with 10% EtOAc in hexanes) to give steroid **18** (556 mg, 92%): ^{1}H NMR (400 MHz, $CDCl_3$) δ 4.65-4.62 (m, 2H), 3.82 (s, 1H), 3.36 (s, 3H), 3.33 (s, 3H), 3.22 (t, J = 7.6 Hz, 1H), 2.00-0.71 (m, 22H), 0.78 (s, 3H), 0.74 (s, 3H); ^{13}C NMR (100 MHz, $CDCl_3$) δ 94.5, 90.8,71.6, 57.8, 55.1, 54.4, 51.3, 42.9, 39.7, 38.1, 35.9, 35.3, 33.6, 32.8, 31.5, 28.5, 27.6, 26.3, 23.2, 20.4, 11.6, 11.4.

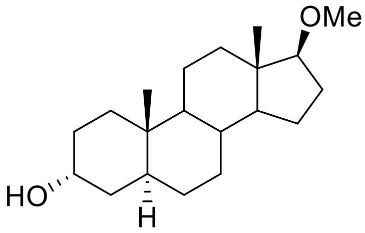

(3α,17β)-3-[[(1,1-Dimethylethyl)dimethylsilyl]oxy]-17-methoxyandrostan-3-ol (19, YX03). To a solution of steroid **18** (556 mg, 1.6 mmol) in methanol (30 mL) was added acetyl chloride (2 mL) at room temperature. After 2 h, water (20 mL) was added and the product was extracted into dichloromethane (100 mL x 2). The organic layer was washed with brine (50 mL x 3). The solvent was removed and the residue was purified by flash chromatography (silica gel, eluted with 5-20% EtOAc in hexanes) to give steroid **19**,**YX03** (477 mg, 97%): mp 166-167 °C; ^{1}H NMR (400 MHz, $CDCl_3$) δ 4.05-4.04 (m, 1H), 3.35 (s, 3H), 3.24 (t, J = 8.2 Hz, 1H), 2.05-0.72 (m, 23H), 0.79 (s, 3H), 0.75 (s, 3H); ^{13}C NMR (100 MHz, $CDCl_3$) δ 90.9, 66.5, 57.8, 54.4, 51.3, 42.9, 39.1, 38.1, 36.1, 35.9, 35.3, 32.2, 31.6, 29.0, 28.4, 27.7, 23.3, 20.4, 11.6, 11.2
